# Sensor‐integrated brain‐on‐a‐chip platforms: Improving the predictive validity in neurodegenerative research

**DOI:** 10.1002/btm2.10604

**Published:** 2023-10-18

**Authors:** Sarah Spitz, Silvia Schobesberger, Konstanze Brandauer, Peter Ertl

**Affiliations:** ^1^ Faculty of Technical Chemistry Vienna University of Technology Vienna Austria; ^2^ Present address: Department of Mechanical Engineering and Biological Engineering Massachusetts Institute of Technology Cambridge Massachusetts USA

**Keywords:** microfluidics, microphysiological systems, neurodegenerative diseases, non‐invasive monitoring, organ‐on‐a‐chip technology, sensors

## Abstract

Affecting millions of individuals worldwide, neurodegenerative diseases (NDDs) pose a significant and growing health concern in people over the age of 60 years. Contributing to this trend are the steady increase in the aging population coupled with a persistent lack of disease‐altering treatment strategies targeting NDDs. The absence of efficient therapeutics can be attributed to high failure rates in clinical trials and the ineptness of animal models in preceding preclinical studies. To that end, in recent years, significant research effort has been dedicated to the development of human cell‐based preclinical disease models characterized by a higher degree of predictive validity. However, a key requirement of any in vitro model constitutes the precise knowledge and replication of the target tissues' (patho‐)physiological microenvironment. Herein, microphysiological systems have demonstrated superiority over conventional static 2D/3D in vitro cell culture systems, as they allow for the emulation and continuous monitoring of the onset, progression, and remission of disease‐associated phenotypes. This review provides an overview of recent advances in the field of NDD research using organ‐on‐a‐chip platforms. Specific focus is directed toward non‐invasive sensing strategies encompassing electrical, electrochemical, and optical sensors. Additionally, promising on‐ and integrable off‐chip sensing strategies targeting key analytes in NDDs will be presented and discussed in detail.

**Translational Impact Statement**

Insufficient predictive validity of animal‐based disease models has restricted the progress in clinical trials targeting neurodegenerative diseases (NDDs). While significant efforts have been made in the replication of intricate micro‐tissue analogs in vitro using OoC technology, these models still fail to integrate non‐invasive sensing strategies, a prerequisite to effective system monitoring. This study provides a comprehensive overview of promising on‐ and off‐chip sensing strategies applicable to NDD research to advance the translational value of microphysiological systems in preclinical settings.

## 1 ORGAN‐ON‐A‐CHIP TECHNOLOGY AS AN IDEAL TOOL TO STUDY NEURODEGENERATIVE DISEASES

### 1.1 Neurodegenerative diseases and the need for advanced in vitro models

Neurodegenerative diseases (NDDs) are a heterogeneous group of disorders characterized by the progressive degeneration of the structure and function of the central or peripheral nervous system paralleled with pathological protein alterations. NDDs can be classified according to their primary clinical features (e.g., dementia, parkinsonism), the anatomic distribution of neurodegeneration, as well as the type of molecular abnormality.^1^ The most common NDDs are amyloidoses, tauopathies, α‐synucleinopathies, and TDP‐43 proteinopathies with Alzheimer's disease (AD), Parkinson's disease (PD), and amyotrophic lateral sclerosis (ALS) constituting the most common representatives.^1^, ^2^ Worldwide, more than 55 million people are affected by dementia, a clinical feature associated with NDDs and an umbrella term used to describe symptoms, including loss of memory and impaired thinking abilities.^3^ According to the World Health Organization, in 2019 alone, the global economic burden associated with dementia, which in 60%–70% of all cases is linked to AD, was estimated at 1.3 trillion US dollars.^4^ As age constitutes the primary risk factor for most NDDs, the socioeconomic burden related to this heterogeneous group of disorders is expected to rise dramatically with an increase in the elderly population (>60 years), which is predicted to double by 2050.^5^ The treatment of NDDs, however, remains largely symptomatic due to the lack of effective treatment options, caused by above‐average high failure rates of promising drug candidates (>85%) in clinical trials.^6^, ^7^ In fact, the monoclonal antibodies Aducanumab (2021) and Lecanemab (2023) are the first two molecules to be approved for treating AD by the Food and Drug Administration in 20 years.^8^, ^9^ In the case of Huntington's disease (HD) and PD, in turn, the lack of neuroprotective and disease‐modifying treatment strategies persists to the present day, restricting their treatment to symptom management.^10^, ^11^ Next to inadequate preclinical studies, one explanation for the low success rate of drug candidates in clinical trials is the insufficient predictive validity of current preclinical animal‐based disease models. Interestingly, despite their often robust replication of NDD‐associated phenotypes, some studies have even become representative examples of how convincing in vivo research data effectively becomes insignificant without a profound knowledge of inter‐species differences.^12^, ^13^, ^14^, ^15^, ^16^, ^17^, ^18^, ^19^ For example, semagacestat a γ‐secretase inhibitor reduced amyloid‐β (Aβ) production in animal models of AD, whereas it exacerbated cognitive and functional deficits and caused excessive numbers of skin cancers in trial settings.^16^ Alternatively, AN1792, a vaccine that prevented plaque burden in AD mice models resulted in meningoencephalitis in 6% of trial participants, likely due to a T‐cell‐mediated autoimmune response.^16^, ^20^ One underlying reason is the uniquely human nature of NDDs as well as their multifactorial origin. In fact, only a fraction of NDDs (e.g., <5% in AD, around 15% in PD) are caused by familial mutations, whereas the majority are sporadic.^21^, ^22^ However, most NDD animal models are transgenic, and while replicating critical pathophysiological phenotypes, fail to fully emulate the clinicopathological or genetic complexities of human NDDs respectively.^16^, ^17^, ^18^, ^19^, ^23^

In summary, there has been an imminent need for alternative disease models that provide higher predictive validity and, thus, better trial outcomes. The search has gathered significant momentum with a recent Food and Drug Administration regulation stating that human clinical trials no longer necessitate animal testing data, paving the way for advanced in vitro modeling strategies in pre‐clinical trials.^24^ One modeling strategy particularly suitable to address the need for advanced in vitro models is OoC technology. OoC technology sets out to emulate (patho‐)physiological tissue niches that account for organotypic cellular arrangements as well as biophysical stimuli (e.g., compression, stretch, shear stress) via spatial and temporal control over cellular microenvironments. Furthermore, its high flexibility in design, material, and device function, coupled with the capability of non‐invasive monitoring via integrated microsensors, allows for the generation of analytically accessible in vitro models, a prerequisite to effective system understanding and control. At the same time, experimental costs can be kept low by the miniaturization of culture chambers and maintenance of high throughput compatibility. In comparison to conventional in vitro models, OoC technology thus offers three key advantages: (i) spatial and temporal control over cells and biochemical cues, (ii) biophysical stimulation as well as (iii) analytical accessibility.^25^, ^26^, ^27^, ^28^ For these reasons, OoC technology has already led to the successful development of various tissue analogs in vitro, including, among others, models of the human blood–brain barrier (BBB) as well as the central nervous system applicable to the study of key pathological mechanisms and the identification of new drug targets.^27^, ^29^, ^30^, ^31^

### 1.2 Recent advances in organ‐on‐a‐chip models for neurodegenerative disease research

Most microfluidic studies on NDDs to date have been dedicated to unraveling the mechanisms behind the uptake, transport, and transmission of aberrant proteins by utilizing the beneficial combination of cellular compartmentalization and optical accessibility obtained within microfluidic setups. While these predominately 2D studies have provided valuable insights into key mechanisms underlying NDD onset and progression, most of the studies (around 80%) have employed over‐simplified models with non‐human cell sources (70%).^26^, ^27^, ^32^ A handful of studies, however, have started to utilize the broader toolset of OoC technology to increase physiological complexity and mimicry and, thus, potential predictive validity. The latter are presented in the following section.

**FIGURE 1:**
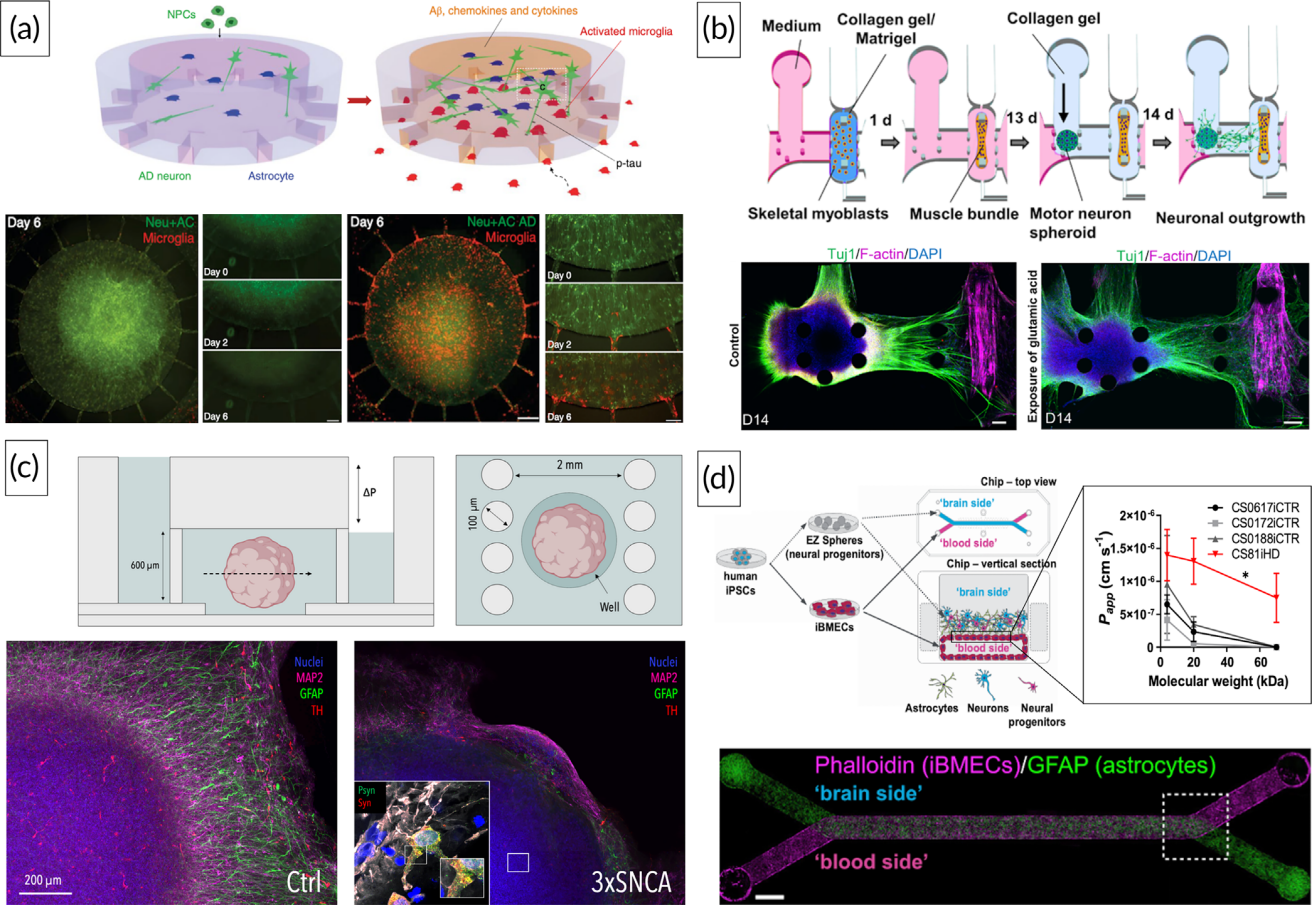
Compartmentalized OoC model for the emulation of Alzheimer's disease (AD)‐associated phenotypes (upper panel). Lower panel illustrates microglial infiltration upon co‐culture with AD‐specific neurons and astrocytes. Reproduced with permission from Park et al.,^33^ 2018, Nature Research (a). Bioengineered neuromuscular junction for the emulation of amyotrophic lateral sclerosis (ALS) phenotypes (upper panel). Representative images of healthy and ALS‐specific neuromuscular junctions (lower panel). Reproduced with permission from Osaki et al.,^38^ 2018, Science (b). OoC platform for the long‐term cultivation of control and patient‐specific (3xSNCA) human midbrain organoids (upper panel). Representative images of control and patient‐specific organoids at D60 of differentiation including the presence of aggregated α‐syn in 3xSNCA organoids (lower panel). Reproduced with permission from Spitz et al.,^31^ 2022, BioRxiv (c). Membrane‐based platform for the assessment of impaired barrier integrities using Huntington's disease (HD)‐specific cells. Representative image of the OoC platform (lower panel). Reproduced with permission from Vatine et al.,^39^ Copyright 2019, Elsevier (d).

To study microglial involvement in AD, Park et al. utilized a compartmentalized device consisting of two circular chambers interconnected via radially organized microchannels (Figure 1a). 3D co‐cultivation of human AD‐specific neurons, astrocytes, and microglia led to the emulation of key pathological phenotypes of the proteinopathy in vitro, including Aβ aggregation, accumulation of phosphorylated tau, as well as microglial recruitment and neurodegeneration.^33^ Bhattacharyya et al. introduced Matrigel® into a conventional Campenot‐like chamber to study the correlation between mitochondria‐associated endoplasmic reticulum membranes, which have been shown to be upregulated in AD, and β‐secretase activity. Downregulating mitochondria‐associated endoplasmic reticulum membranes attenuated β‐secretase cleavage of the palmitoylated amyloid precursor protein and reduced axonal release of Aβ, demonstrating a new route for therapeutic intervention.^34^ Shin et al. investigated the effect of pathological Aβ on vascular integrity by co‐culturing neurons expressing the amyloid precursor protein with familial AD mutations adjacent to a single vascular lumen in a parallelly aligned microfluidic setup. The authors reported a significant increase in vascular permeability mediated by a reduction in the expression of the tight junction proteins claudin‐1 and claudin‐5 as well as the adherens junction protein VE‐cadherin. In addition, increased levels of MMP2 and reactive oxygen species (ROS) were detected in the abluminal side of the vessel when co‐cultured with AD‐specific neurons. Finally, a phenotypic rescue was observed when treating neurons with BACE1, an inhibitor of the β‐secretase enzyme.^35^ Next to AD, significant progress has been made in the emulation of PD‐specific phenotypes using microfluidic platforms. Culturing patient‐derived dopaminergic neurons carrying a familial PD mutation (LRRK2) in a hydrogel within a commercially available microfluidic platform led to the replication of robust pathological phenotypes in vitro, including decreased neuronal differentiation, impaired neuronal complexity, increased neuronal death as well as altered mitochondrial morphology. High‐content image analysis further revealed that treatment with an LRRK2 inhibitor resulted in phenotypic rescue, with the latter being linked to the genetic background of the cells rather than the pathologic mutation itself.^36^ Spitz et al. recently demonstrated the beneficial effects of cultivating midbrain organoids using OoC platforms, including improved nutrient supply and reduced necrotic core formation. Key pathological phenotypes such as reduced populations of dopaminergic neurons, impaired glial differentiation, aggregated α‐synuclein (α‐syn), and compromised mitochondrial phenotypes were observed upon comparing patient‐specific organoids carrying a triplication mutation of the α‐syn gene with control organoids at 60 days of differentiation (Figure 1c). Furthermore, rescue effects in mitochondrial and neurodegenerative phenotypes were observed after the treatment of patient‐specific organoids with the repurposed compound 2‐hydroxypropyl‐β‐cyclodextrin.^31^ Pediaditakis et al., on the other hand, employed a membrane‐based platform to investigate the role of the BBB in PD by co‐culturing human dopaminergic neurons, astrocytes, pericytes, microglia, and microvascular endothelial cells under dynamic conditions. Exposing the neuronal compartment to fibrillar α‐syn led to pathology‐associated phenotypes, including the presence of phosphorylated α‐syn, mitochondrial impairment, neuroinflammation, and compromised barrier function.^37^ Vatine et al. employed a similar platform (Figure 1d) to assess patient‐specific differences in barrier permeability in the context of HD.

Brain microvascular endothelial cells derived from an HD patient displayed significantly higher permeabilities toward fluorescently labeled dextrans of varying sizes when compared to endothelial cells of three healthy controls.^39^ Finally, by combining spheroids of patient‐derived motor neurons with an engineered muscle bundle on chip, Osaki et al. emulated pathological phenotypes associated with the neurodegenerative disorder ALS (Figure 1b). ALS motor units displayed fewer muscle contractions, motor neuron degeneration as well as increased muscle apoptosis. Phenotypic rescue in muscle contraction was reported upon co‐administration of the two drugs rapamycin and bosutinib via an additional endothelial barrier integrated into the platform.^38^ In summary, the abovementioned studies, albeit limited in number, clearly demonstrate the vast potential of OoC technology in NDD research, including the ability to study key pathological mechanisms underlying NDDs as well as to identify novel therapeutic intervention strategies in vitro. It has to be noted, however, that while current microphysiological NDD models have emulated critical pathological phenotypes, they still lack essential elements required to fulfill a high degree of (patho‐)physiological mimicry, including, among others, the co‐presence of perfusable vascular networks of the BBB, the meningeal lymphatics as well as tissue‐specific cellular subtypes and immune cells (e.g., T‐cells). For example, decreased cerebral blood flow has been linked to accelerated cognitive decline as well as increased risk of dementia, and has been considered one of the earliest pathological events in the onset of NDDs (e.g., AD).^40^, ^41^ Furthermore, several studies have reported dysfunction of the meningeal lymphatics, a key drainage route responsible for the clearance of molecules, immune cells, and cellular debris in aging as well as neurodegeneration.^42^ In other words, accounting for both pathological phenotypes in vitro would open up the possibility to investigate novel therapeutic intervention strategies targeting NDDs. Finally, there has been a growing body of evidence implicating T‐cells in the pathogenesis of NDDs, rendering their consideration essential in the design of in vitro models.^43^ However, significant progress has been made in the microphysiological recapitulation of these constituents individually (e.g., engineered networks of the BBB, the lymphatic system, T‐cell perfusion), providing an ideal basis for future NDD models.^27^, ^29^, ^44^, ^45^, ^46^

**FIGURE 2:**
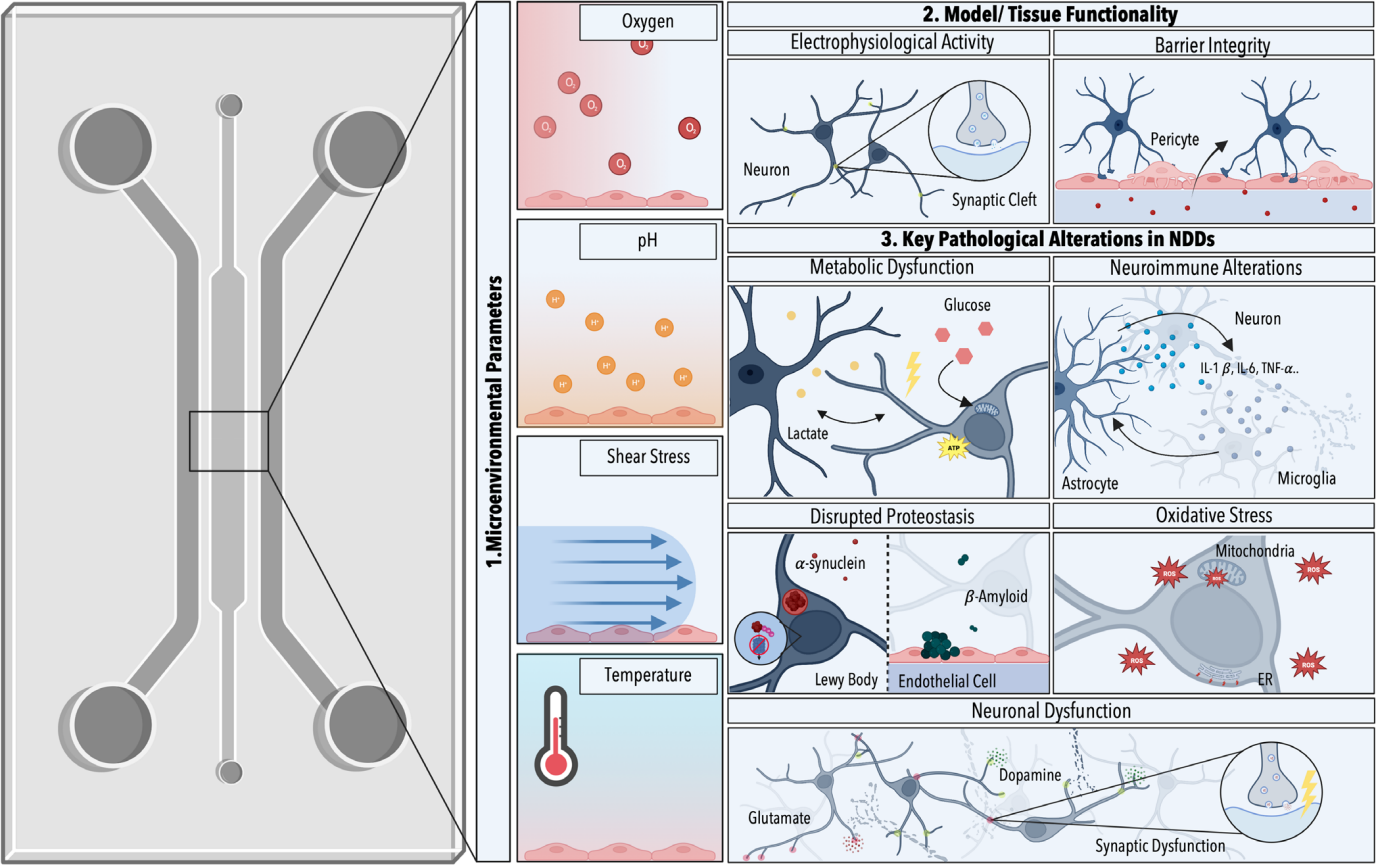
Schematic highlighting key parameters/analytes for effective system control in neurodegenerative disease (NDD)‐based in vitro models: (1) microenvironmental parameters, (2) model‐specific parameters for validating microtissue functionality, and (3) NDD hallmark‐specific mechanisms/analytes. Illustration created with BioRender.com.

Furthermore, to meet the need for predictive NDD in vitro models, effective microphysiological system monitoring is paramount. This is of particular importance considering both the increasing complexity and potential variability of microtissue‐analogs as well as the occurrence of dynamic changes within NDD‐associated phenotypes. Specifically, effective system monitoring will require time‐resolved knowledge of (i) the cellular microenvironments, including, for example, shear stress, pH, and oxygen tension, (ii) tissue‐specific functionality, as well as (iii) pathological alterations. While NDDs display individual and disease‐specific traits they share a range of common hallmarks, applicable to non‐invasive monitoring. Depending on the study shared traits among NDDs have been summarized into a handful of interconnected hallmarks including, metabolic dysfunction, synaptic and neuronal network dysfunction, disrupted proteostasis, neuroinflammation as well as oxidative stress (Figure 2). For a comprehensive overview of NDD‐associated hallmarks please refer to Wilson et al. and Bloomingdale et al.^47^, ^48^

Thus, to improve analytical accessibility, a prerequisite to effective system control, OoC models will need to incorporate multi‐parametric sensing strategies capable of non‐invasive monitoring. While an increasing number of studies are documenting the utilization of sensor‐integrated OoC platforms, however, most of the published manuscripts to date provide mere proof of principles that fail to emulate (patho‐)physiological tissue niches in vitro.

## 2 SENSOR‐INTEGRATED ORGAN‐ON‐A‐CHIP PLATFORMS TO STUDY NEURODEGENERATIVE PROCESSES IN VITRO

**FIGURE 3:**
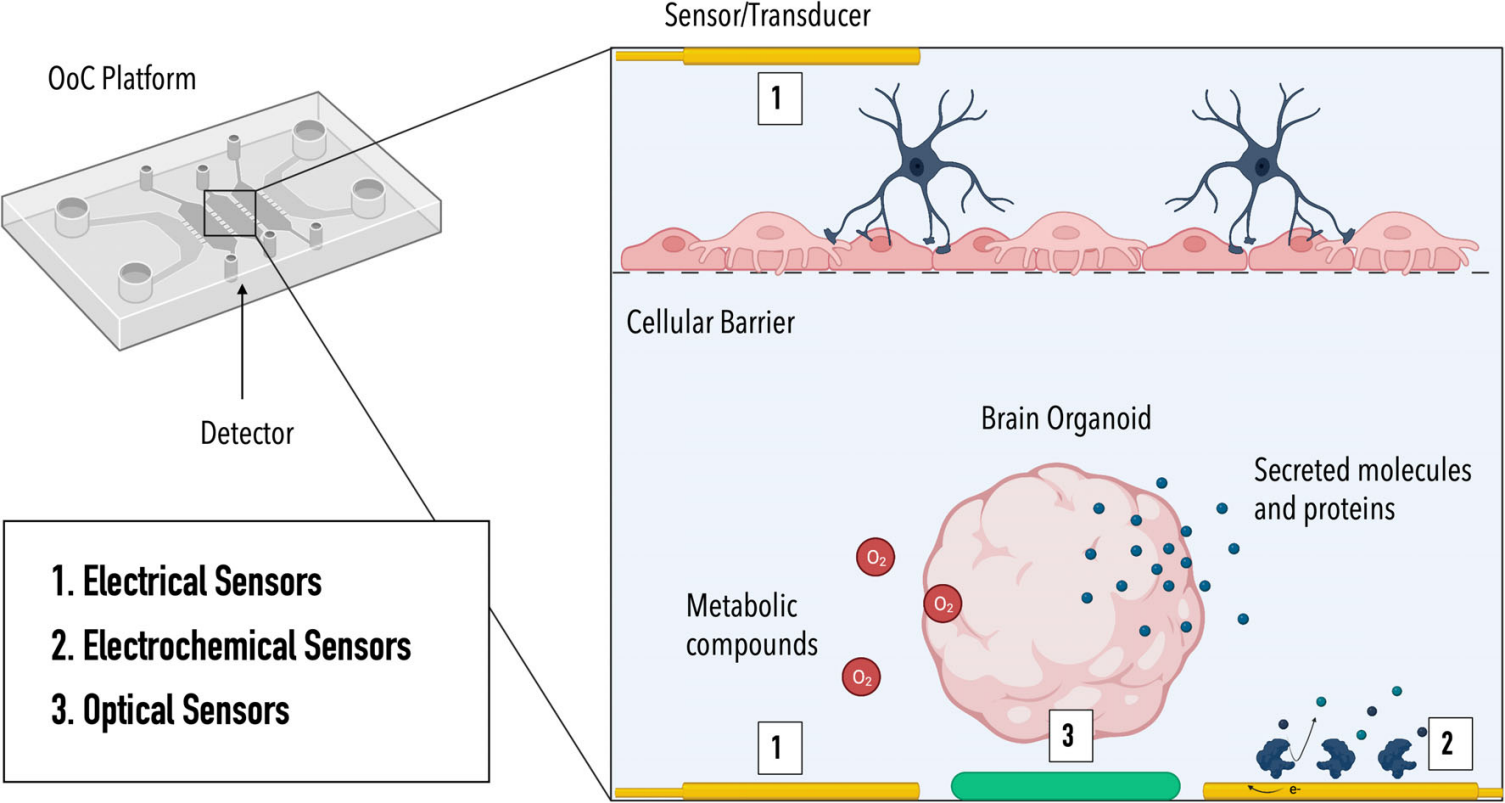
Graphical representation of the three sensing strategies employed in OoC technology: (1) electrical sensors, (2) electrochemical sensors, and (3) optical sensors. Illustration created with BioRender.com.

In general, sensing strategies in OoC technology can be separated into (i) electrical, (ii) electrochemical, and (iii) optical approaches (Figure 3). Due to their ease of integration and suitability for long‐term cultures, electrical sensors constitute the predominant sensing strategy within microphysiological systems, providing information about key parameters such as barrier integrity, electrophysiological activity, and mechanical strain.^44^, ^49^, ^50^, ^51^, ^52^, ^53^, ^54^ Electrochemical sensors, on the other hand, employ an electrode surface to convert electrochemical reactions/interactions of an analyte into an electrical signal. Depending on the measurement mode, changes in current (amperometry, voltammetry), potential, impedance, or conductivity are detected. To enhance specificity, electrochemical biosensors employ so‐called biorecognition elements, including antibodies, aptamers, and enzymes, that specifically interact with the analyte of interest.^55^ To deduce information on the status of an OoC system, optical sensors rely on the detection of changes in optical properties, including absorption, scattering, and luminescence.^56^, ^57^, ^58^ Optical sensors hold great promise in OoC systems due to their versatility, non‐invasive nature, and seamless integration.^52^ A prerequisite for the integration of optical sensors such as luminescence‐ or absorbance‐based sensors into OoC devices is the optical accessibility/transparency of the device.^59^ While the most common optical sensors in OoC technology are oxygen sensors,^60^, ^61^, ^62^, ^63^, ^64^, ^65^, ^66^ optical sensing strategies have also been employed for the reliable detection of pH, secreted molecules (e.g., cytokines, insulin)^67^, ^68^ as well as morphological changes.^57^ The following section will provide an overview of promising on‐ and off‐chip sensing strategies applicable to monitoring critical microenvironmental parameters and key analytes in NDD research. For a comprehensive overview of all relevant on‐ and off‐chip sensing strategies, including commercially available sensors, please refer to Supporting Information Tables S1–S4.

### 2.1 Monitoring microenvironmental parameters to establish reliable in vitro models

Effective system control, a prerequisite to reproducible cultivation conditions and thus reliable scientific outcomes, necessitates knowledge of the emulated microenvironment, including information on dissolved oxygen levels, pH, temperature, flow rates, and shear stress.

Oxygen availability is not only essential for optimal energy metabolism but exhibits significant variation across different regions within the human body.^69^ As such, precise fine‐tuning of dissolved oxygen levels is required for the establishment of microphysiological models. It has to be noted that oxygen levels within microfluidic platforms strongly depend on the gas permeability of the used materials (e.g., device, tubing) as well as the employed cultivation setups (e.g., static versus dynamic).^64^, ^70^ While this correlation is beneficial for modulating oxygen gradients in microfluidic devices, it necessitates precise control using integrated sensors. Next to unphysiological oxygen levels, fluctuations in pH can have detrimental effects on cellular homeostasis. Consequently, both parameters have already been successfully monitored in OoC platforms, with a range of different sensing strategies being available for each analyte. Müller et al., for example, integrated luminescent‐based oxygen and pH sensors into a microfluidic platform to monitor drug‐mediated metabolic changes of a human lung carcinoma epithelial‐like cell line (Figure 5h).^71^ For further information on oxygen sensing strategies, please also refer to Section 2.3.1. As shear stress intricately regulates tissue‐specific functions at the cellular level, accurate monitoring in microfluidic devices is essential. Booth et al., for example, integrated a flow sensor array that utilizes a thermal conductivity detector to monitor shear stress levels within microfluidic channels.^72^ Several published and commercially available flow sensors have been reported. For further details on flow sensors, please refer to Huang et al.^73^ In summary, sensors for the most critical microenvironmental parameters have already been successfully integrated into OoC platforms and are readily available. To illustrate, Zhang et al. reported a multiplexed sensing approach by interconnecting two OoC platforms to two individual sensing units. Using this setup, the authors were able to simultaneously monitor the microenvironmental parameters oxygen, pH, temperature, and flow rate on a physical/chemical module, while downstream recording of secreted biomarkers was achieved using an electrochemical sensing unit.^74^

### 2.2 On‐ and off‐chip sensing strategies to assess tissue functionality

In addition to establishing defined cultivation conditions, ensuring the reproducibility of in vitro models and, consequently, research outcomes necessitates a thorough evaluation of microtissue functionality. While the specific parameters to be monitored depend on the type of emulated tissue as well as its intricacy, two fundamental functions take center stage when modeling NDDs: maintaining BBB integrity and overseeing neuronal activity (Figure 2). Monitoring strategies applicable to these two functionalities are discussed below.

#### 2.2.1 Barrier integrity

**FIGURE 4:**
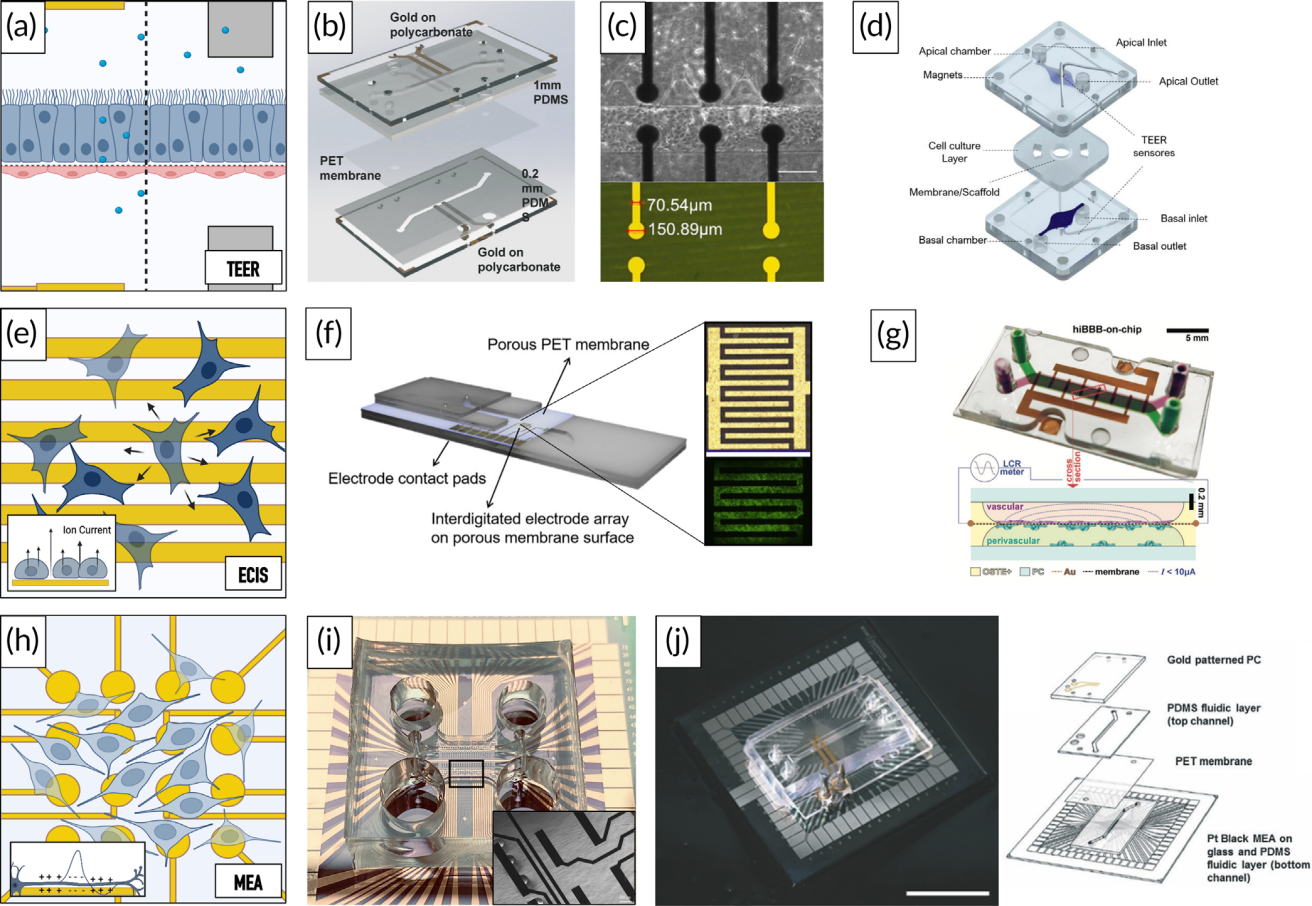
Graphical representation of microfluidic trans‐epithelial/endothelial electrical resistance (TEER) setups (a). Representative examples of TEER‐integrated OoC platforms using either gold‐sputtered (b, c) or inserted Ag/AgCl electrodes (d). Reproduced with permission from Henry et al,^88^ 2017, The Royal Society of Chemistry (b), Palma‐Florez et al.,^95^ 2023, Springer Nature (c), and Zoio et al.,^90^ 2021, MDPI (d). Graphical representation of electrical cell‐substrate impedance sensing (ECIS) measurements (e). Representative examples of membrane‐integrated ECIS sensors in PDMS– (f) and OSTE+ (g)‐based OoC platforms. Reprinted with permission from Schuller et al.,^97^ 2020, Elsevier (f), and Matthiesen et al.,^50^ 2021, Wiley (g). Graphical representation of multi‐electrode array (MEA) measurements. (h) Representative examples of a planar MEA integrated into the media channels of an OoC platform for the recording of electrophysiological activities of 3D midbrain tissues. Inset shows flow‐directed neurite outgrowth onto electrodes (i) and a multi‐sensor integrated MEA and TEER platform (j). Reprinted with permission from Spitz et al.^31^ (i) and Maoz et al.,^49^ 2017, Royal Society of Chemistry (j). Illustrations created in Biorender.com (a, e, h).

The BBB plays an essential role in maintaining cerebral homeostasis by selectively regulating the transport of molecules from the blood to the cerebrum and vice versa. Impaired barrier integrity has been reported in several NDDs, including AD and PD.^75^ As such, BBB integrity constitutes an essential monitoring parameter providing information on the degree of physiological mimicry, and the development of pathological phenotypes, while simultaneously ensuring reliable and reproducible conditions. It has therefore been the main focus in a variety of studies focusing on the development of trans‐epithelial/endothelial electrical resistance (TEER)‐integrated OoC platforms.^39^, ^76^, ^77^, ^78^, ^79^, ^80^, ^81^, ^82^, ^83^, ^84^, ^85^ TEER constitutes a non‐invasive and quantitative method that monitors the change in resistance between two electrodes placed on either side of a semipermeable substrate employed for culturing barrier‐forming cells (Figure 4a). TEER can either be obtained directly by probing at a selected frequency or by fitting impedance spectra data to an adequate circuit model and subsequently extracting the cellular resistance.^86^ Jeong et al., for example, placed two multi‐electrode arrays (4 × 4 – on polycarbonate) opposite each other to monitor the barrier integrity within each compartment of a membrane‐based multiplexed (16‐chamber) murine BBB model. Using real‐time and non‐invasive monitoring, the authors could show the beneficial effect of the microfluidic co‐culture of astrocytes and endothelial cells on barrier integrity pre and post histamine stimulation.^87^ Another study employed a four‐electrode approach, previously reported by Henry et al., to assess the effect of hypoxia on the differentiation of induced pluripotent stem cell (iPSC)‐derived brain microvascular endothelial cells (Figure 4b). The authors could show that iPSC‐derived brain microvascular endothelial cells exposed to hypoxia during differentiation presented significantly higher barrier integrities in co‐culture with primary astrocytes and pericytes as opposed to brain microvascular endothelial cells differentiated under normoxic conditions.^88^, ^89^ Next to sensor‐integrated platforms, TEER values have also been obtained via the insertion of electrodes into the reservoirs/channels of microfluidic chips (Figure 4d).^90^ Using non‐polarizable electrodes (Ag/AgCl), Fengler et al., for example, were able to determine the barrier integrity of their human endothelial cell‐based BBB model in a commercially available microfluidic platform (Organoplate®).^91^ Similarly, Ahn et al. demonstrated improved TEER values under co‐culture conditions as well as with increasing shear stress (0.4–4 dyne/cm^2^) by inserting Ag/AgCl wire electrodes into their triple‐culture BBB model.^92^ While this approach is characterized by its remarkable ease of integration, it must be emphasized that next to inhomogeneous electrical fields resulting from spatial restrictions in electrode placement, increased electrode‐cell distances can result in overestimated TEER values.^51^ Furthermore, potential cytotoxic effects must be considered when using Ag/AgCl electrodes.^93^ Odijk et al. tried to account for the discrepancies between traditional and microfluidic TEER setups through the development and subsequent validation of a mathematical model using experimental data. Using the proposed model, the authors could extrapolate comparable TEER data from their microfluidic gut‐on‐a‐chip device as long as the tissue analog remained in a monolayer regime (first ~70 h). In their study, Odijk et al. further showed that a slight reduction in cell coverage (0.4%) could cause a significant drop (80%) in TEER, underlining the methods' intrinsic sensitivity and, thus, low comparability.^94^ The latter is further reinforced by another study by the authors that demonstrated an 11‐fold decrease in sensitivity upon increasing the temperature from 21 to 37°C, whereas increasing the ion concentration (0.09–0.15 M KCl) decreased the resistance by a factor of 5. As such, next to careful design considerations, a controlled measurement environment is of the utmost importance when conducting TEER measurements.

By placing microelectrodes on either side of a micropillar array, Palma Florez et al. developed a micro‐TEER approach capable of assessing the barrier integrities of vertically arranged endothelial monolayers, as obtained in parallel microfluidic setups (Figure 4c).^95^ A different methodology using voltammetry was reported by Wong et al. In voltammetry, the working electrodes' potential is continuously varied over time in a range characteristic of the analyte's redox reaction. Thereby either oxidation or reduction of an electroactive molecule is induced and subsequently measured. In their study, electrodes were integrated at the bottom of a bilayer microfluidic platform to measure the penetrance of electroactive tracers through a monolayer of endothelial cells.^96^

Another promising alternative to conventional TEER measurements has been developed by Schuller et al., who employed interdigitated electrodes to monitor the integrity of a placental monolayer using electrical cell‐substrate impedance sensing (ECIS) (Figure 4f).^97^ A similar approach was reported by Matthiesen et al., who employed this strategy to monitor changes in the barrier integrity of an iPSC‐derived BBB model (Figure 4g).^50^ In contrast to TEER measurements, ECIS employs an array of small working electrodes combined with a large common counter electrode to monitor local changes in impedance, usually at a pre‐selected frequency (Figure 4e).^51^, ^98^ To illustrate, Liu et al. utilized ECIS to monitor changes in cortical network integrity upon the addition of Aß42 in a compartmentalized microfluidic device modeling AD. Optical accessibility was enabled by the use of transparent indium tin oxide electrodes.^99^

#### 2.2.2 Electrophysiological activity

Monitoring electrophysiological activity has become an indispensable tool for assessing cerebral tissue (dys‐)function since changes in neuronal activity patterns, such as variations in the firing rate and synchronization of neurons, are believed to reflect underlying alterations in cellular structure and functionality.^100^ Imbalances in extracellular ion concentrations or transmembrane potentials, which occur during de‐ and repolarization events of electrically active cells, can be readily detected using voltage‐sensitive electrodes, such as multi‐electrode arrays (MEAs). MEAs comprise hundreds of microelectrodes that enable the spatiotemporal mapping of electrophysiological events (Figure 4h).^101^ Müller et al., for example, demonstrated the vast potential of MEAs by culturing and analyzing networks of cortical neurons on a complementary metal‐oxide semiconductor (CMOS)‐based device containing 26.400 microelectrodes. Using this platform, the authors were able to simultaneously record 1.024 channels providing information on network activities with cellular and subcellular resolution. While the CMOS‐based platform offers superior spatial resolution, its complex fabrication process and optical inaccessibility hamper a broader application.^102^ Using interstitial fluid flow to direct neuronal outgrowth onto planar MEAs, Spitz et al. have recently demonstrated the applicability of 2D MEAs for recording the electrophysiological activity of 3D midbrain organoids in an OoC platform (Figure 4i).^31^

It must be noted, however, that while Au (the most commonly employed material in MEAs) and Pt electrodes are considered biocompatible, their polarizability (charging effects at the electrode surface) may induce large contact impedance that can interfere (i) with impedance measurements, particularly at low frequencies, and (ii) with extracellular field potentials.^51^ Maoz et al. addressed this limitation by increasing the surface area of the electrodes through the deposition of Pt black onto an Au‐MEA in a combined TEER and MEA OoC platform (Figure 4j).^49^ Overall, while planar MEAs can be readily integrated into OoC platforms, they are primarily suitable for 2D applications as close proximity between electroactive cells and electrodes is required for effective use. Substantial progress, however, has been made in the development of 3D MEAs in recent years. Soscia et al., for example, developed a flexible polyimide‐based 3D MEA capable of recording the electrophysiological activity of iPSC‐derived neurons and astrocytes cultivated within a hydrogel matrix over a period of 45 days (Figure 6d).^103^ Shin et al. reported a high‐density multi‐functional MEA, incorporating optical stimulation and drug delivery capabilities using both an LED light source and an integrated microfluidic platform. The authors recorded temporal changes in spontaneous electrophysiological activity of iPSC‐derived cortical neuronal populations over a period of 14 days, assessed the effect of chemical and optical stimulation, and investigated the functional connectivity of compartmentalized neuronal groups.^104^ Despite the intricate fabrication processes associated with 3D MEAs, the latter constitute promising tools for conducting spatial electrophysiological analysis in OoC platforms.

### 2.3 Non‐invasive strategies for monitoring pathological alterations associated with neurodegenerative diseases

#### 2.3.1 Monitoring metabolic dysfunction in vitro

Metabolic dysfunction has been reported in various NDDs including AD, PD, HD as well as ALS.^47^, ^48^ To cover their high energetic demand, neurons rely predominantly on the two metabolites glucose and lactate as well as oxidative phosphorylation.^105^ Consequently, dysfunctions within mitochondria mediated, for example, by aggregated proteins or changes in cytoskeletal dynamics disturb neuronal homeostasis and impair neuronal functionality.^47^, ^48^ To emphasize, two of the genes associated with familial PD: Parkin and PINK1, both encode proteins that are involved in mitochondrial quality control pathways.^106^, ^107^ However, mitochondrial dysfunction also occurs in NDDs with non‐mitochondrial etiology, rendering the two energetic substrates as well as oxygen ideal analytes in monitoring metabolic dysfunction in vitro.

##### Oxygen

Oxygen levels within the brain are maintained within a narrow range that corresponds to region‐specific brain activities.^108^ Hypoxia induces oxidative stress and ROS‐induced DNA damage, leading to accelerated cellular senescence, increased susceptibility to neurological pathologies, and inflammation characterized by the upregulation of TNF and IL‐1.^48^ Oxygen constitutes an important mediator in neuronal and glial differentiation and has been shown to be differentially consumed by individual cells of the cerebrum including neural stem cells, neurons, and astrocytes.^109^ As such, monitoring oxygen levels not only provides valuable insights into cellular populations and metabolic phenotypes but also constitutes an important prerequisite for precisely controlling cellular microenvironments.^110^ In general, either optical or electrochemical sensors can be employed for the detection of oxygen in microfluidic systems. While electrochemical sensors, such as amperometric Clark‐type sensors, constitute the most common oxygen‐sensing strategy, the sensors are restricted by analyte consumption and increased fabrication complexity. Moya et al. addressed this limitation by employing inkjet printing, a low‐cost method that does not require the use of photolithography, to integrate an electrode array onto a membrane within a liver‐on‐a‐chip platform. The sensor was validated using a commercial Clark‐type electrode and was able to detect oxygen gradients within the microfluidic microenvironment.^111^

**FIGURE 5:**
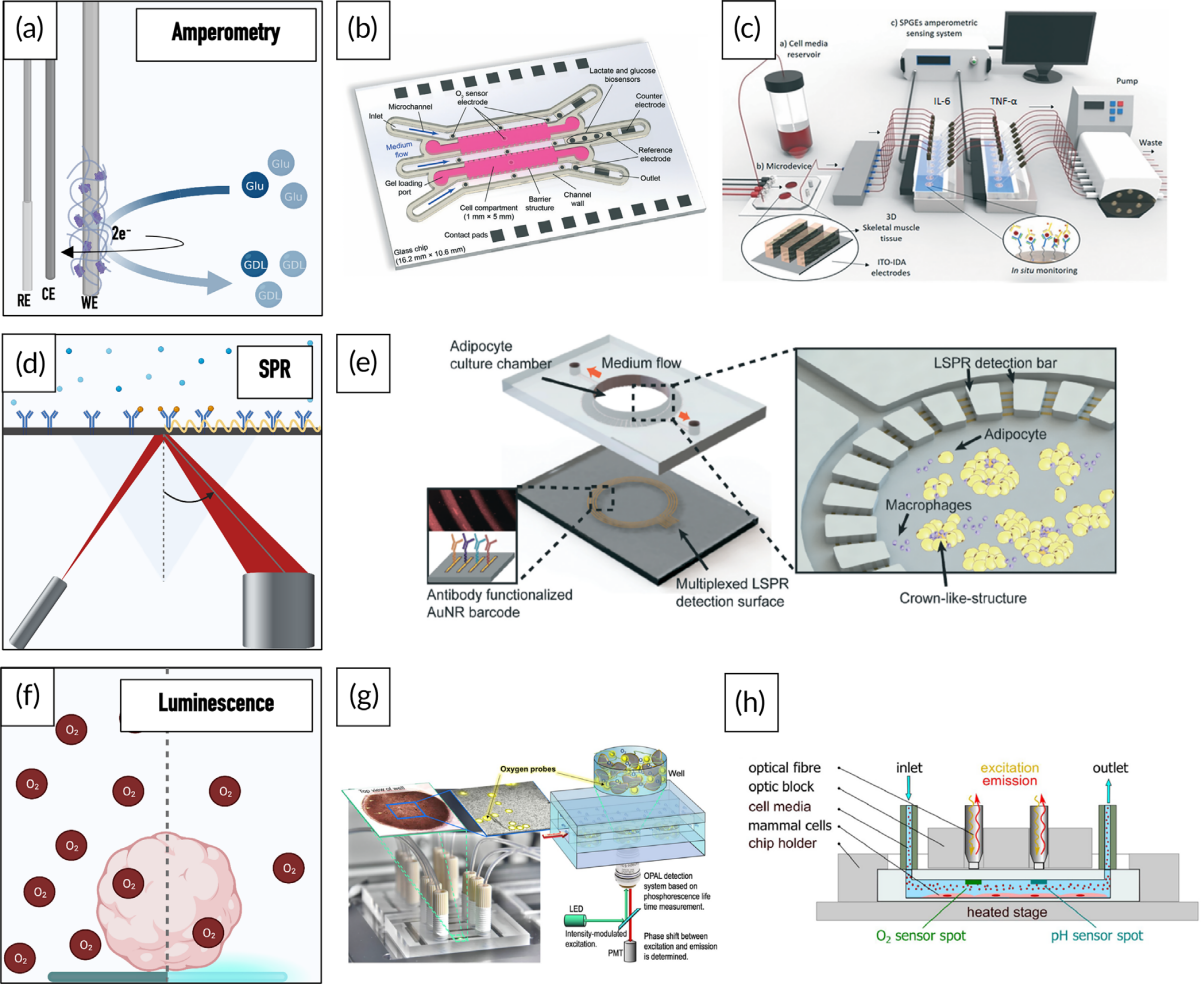
Sensing principle of amperometric measurements of analytes that are enzymatically converted (e.g., glucose [Glu] oxidized by glucose oxidase to gluconolactone [GDL]) using a 3‐electrode setup (a). OoC platform with integrated amperometric oxygen, glucose, and lactate sensors. Reproduced with permission from Dornhof et al.,^118^ 2022, The Royal Society of Chemistry (b). Schematic of a muscle‐on‐a‐chip platform connected to sensing modules equipped with screen‐printed electrodes for the amperometric detection of IL‐6 and TNF‐⍺. Reproduced with permission from Ortega et al.,^133^ 2019, The Royal Society of Chemistry (c). Graphical illustration of the working principles of surface plasmon resonance: Binding of biomolecules to immobilized antibodies leads to a change in the refractive index of the sensor's surface layer (d). Adipose‐on‐a‐chip platform with a nanoplasmonic biosensor for the multiplexed analysis of tissue inflammation markers. Reproduced with permission from Zhu et al.,^52^ 2018, The Royal Society of Chemistry (e). A schematic illustration of a luminescence sensor based on fluorescence quenching (f). Microfluidic device with integrated luminescence‐based oxygen sensor particles suspended in a collagen matrix with hepatocytes. Reproduced with permission from Gehre et al.,^63^ 2020, Springer Nature (g) Graphical illustration of a microfluidic setup with integrated oxygen and pH sensor spots for simultaneous monitoring of respiration and acidification rates. Reproduced with permission from Müller et al.,^71^ 2021, Elsevier (h). Illustrations created in Biorender.com (a, d, f). CE, counter electrode; RE, reference electrode; WE, working electrode.

Optical sensors primarily rely on luminescence to provide information on oxygen concentrations in vitro. In detail, the emission of a luminescent sensor is quenched by triplet oxygen, the most common allotrope, in a partial pressure‐dependent manner (Figure 5f). Read‐out is focused either on monitoring decreases in fluorescent intensity or signal lifetime.^112^, ^113^ Matsumoto et al. used an intensity‐based luminescent sensor film to spatially monitor the cellular respiration of HepG2 cells. By controlling the oxygen gradients and partial pressures via the applied flow rates, the researchers were able to emulate periportal and perivenous hepatic like‐zones in their OoC device.^65^ While intensity‐based read‐outs can be performed using a conventional fluorescence microscope, it should be noted that measurements can be influenced by several factors, such as inconsistencies in the measuring system and the sensor, as well as issues including photobleaching and dye leaching. Lifetime‐based sensors, on the other hand, record the time that passes between the excitation of a fluorescent indicator dye and the subsequent emission of a photon, thereby providing a more robust read‐out. Zirath et al. have previously shown that lifetime‐based optical oxygen sensors can easily be integrated into microfluidic platforms and can provide valuable information on the formation of oxygen gradients, cellular viabilities as well as cell‐specific respiration profiles.^58^, ^64^ Furthermore, the sensor was successfully integrated into a midbrain‐on‐a‐chip platform for long‐term studies, revealing significant differences in the oxygen demand between control and PD‐specific midbrain organoids, as well as upon treatment with a repurposed compound.^31^ Furthermore, several commercially available oxygen sensors have been reported (Table S4). Super et al., for example, employed a commercially available optical oxygen sensor for the real‐time monitoring of oxygen uptake rates of embryonic stem cells.^114^ Gehre et al. embedded commercially available oxygen sensor beads into a collagen matrix together with hepatocytes (Figure 5g) to continuously monitor cellular respiration in 3D constructs for a duration of 6 days.^63^

##### Glucose

**FIGURE 6:**
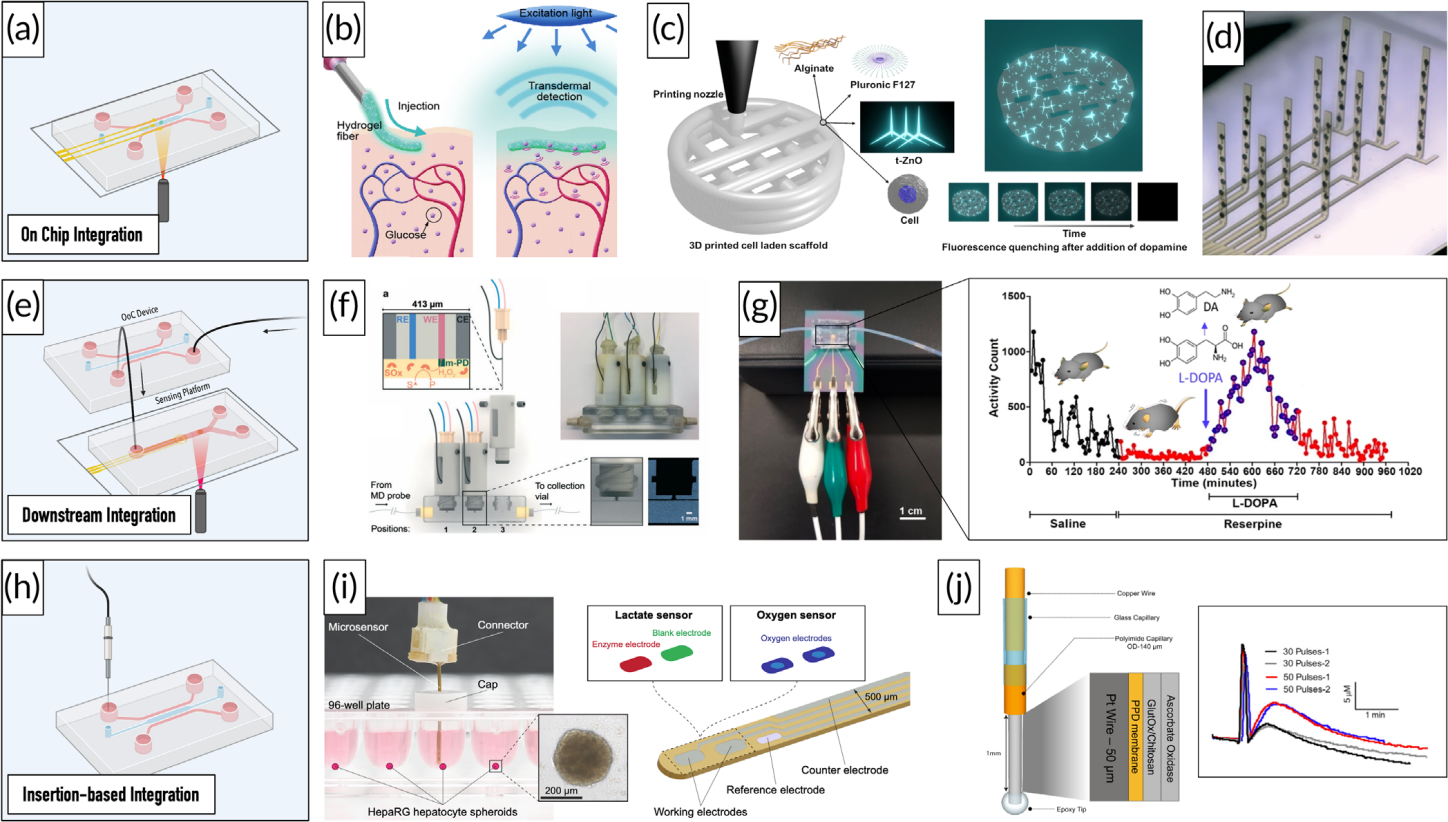
Overview of potentially integrable on‐ and off‐chip sensing strategies. Schematic illustration of on‐chip integration (a). A biocompatible glucose‐sensitive hydrogel to optically monitor glucose subcutaneously for up to 140 days in mice. Reproduced with permission from Heo et al.,^123^ 2011, PNAS (b). An optical sensing approach based on tetrapodal‐shaped‐ZnO microparticles printed into an alginate/Pluronic F127 gel to detect dopamine via fluorescence quenching. Reproduced with permission from Li et al.,^145^ Copyright 2023, Elsevier (c). Polyimide‐based MEAs for the 3D‐resolved electrophysiological recording of neurons suspended in a hydrogel matrix. Reproduced with permission from Soscia et al.,^103^ 2020, The Royal Society of Chemistry (d). Illustration of a downstream sensor integrated setup (e). Needle‐based electrodes modified with analyte‐specific enzymes integrated into a 3D‐printed housing to monitor the microdialysate of brain injury patients. Reproduced with permission from Samper et al.,^163^ 2019, The Royal Society of Chemistry (f). Electrochemical microfluidic sensor for detecting dopamine levels of reserpine‐treated mice in cerebrospinal fluid. Reproduced with permission from Senel et al.,^146^ Copyright 2020, American Chemical Society (g). Illustration of insertion‐based integration approaches (h). Combined lactate and oxygen microsensor for monitoring metabolic changes in supernatants. Reproduced with permission from Weltin et al.,^127^ Copyright 2016, Elsevier (i). Functionalized microelectrode for enzymatic glutamate detection in vivo. Glutamate levels increase as a response to electrical stimulation. Reproduced with permission from Ganesana et al.,^164^ Copyright 2019, Elsevier (j). Illustrations created in Biorender.com (a, e, h).

Glucose constitutes the primary energy source of the human brain and tight regulation of its metabolism is critical for physiological brain functioning.^115^ As described above, mitochondrial dysfunction is a common pathological phenotype among NDDs, resulting in a compensatory shift from the tricarboxylic acid cycle and oxidative phosphorylation to aerobic glycolysis.^116^ Furthermore, reduced glucose uptake, as well as region‐specific changes in its metabolism, have been reported in NDDs, rendering its monitoring crucial in the context of NDD research.^116^ Electrochemical glucose sensors have already been successfully integrated into OoC systems and employed in multisensor‐integrated platforms (Tables S1–S2). The sensors utilize amperometry to transduce signals from enzymatic reactions that occur at the surface of functionalized biosensors. To illustrate, the enzyme glucose oxidase catalyzes the oxidation of glucose to hydrogen peroxide and D‐glucono‐δ‐lactone (Figure 5a). By applying a potential that oxidizes the by‐product hydrogen peroxide, thus, conclusions on glucose concentrations can be drawn.^61^ Misun et al. integrated enzyme‐based amperometric lactate and glucose sensors into a hanging‐drop chip for monitoring spheroids derived from human colon carcinoma cells. While the combined sensing approach enabled real‐time measurements, its sensitivity was limited to one day.^117^ In general, spatial separation of oxidase‐based sensors from both the cultivation chamber and oxygen sensors is favorable due to the formation of the cytotoxic byproduct hydrogen peroxide and the enzyme's oxygen consumption.^53^ Recently, Dornhof et al. demonstrated an OoC platform with integrated sensors for monitoring oxygen, glucose, and lactate levels of breast cancer stem cell‐derived spheroids embedded in Matrigel®. Glucose and lactate sensors were integrated downstream of the main compartment, whereas oxygen sensors were incorporated into cell cultivation chambers and medium channels. Continuous real‐time monitoring was shown for 1 week and its applicability for studying drug responses was presented by the administration of doxorubicin, a chemotherapeutic drug, and antimycin A, a metabolism‐altering drug (Figure 5b).^118^ Weltin et al. combined pH and oxygen sensors in and close to the cultivation chamber with glucose and lactate sensors integrated downstream of the cell compartment. Using this approach, the authors successfully monitored the metabolic shift of T98G human brain cancer cells as a response to the glucose transport blocker Cytochalasin B.^119^ Similarly, McKenzie et al. developed a Pt screen‐printed electrode comprised of a glucose sensor, a lactate sensor, an oxygen sensor, a pH sensor as well as a reference electrode. Real‐time monitoring of macrophages using stopped‐flow perfusion revealed glucose and oxygen consumption as well as lactate acid secretion; however, sensor stability studies of the enzyme‐based sensors demonstrated considerable signal loss with each week of dry storage. Complete sensitivity loss was observed for the lactate sensor after 2 weeks.^120^ In general, it should be noted that enzyme‐based sensors often require re‐calibration, particularly during long‐term cultivations, due to enzyme degradation.^53^ To address this issue, Bavli et al. developed a connected chip unit with incorporated enzyme‐based lactate and glucose sensors, facilitating the required washing and recalibration steps between each round of measurements. By integrating oxygen probes into 3D HepG2/C3A aggregates, the authors could provide real‐time data on minute shifts from oxidative phosphorylation to anaerobic glycolysis, an indicator of early mitochondrial stress over a period of 28 days.^61^ Giménez‐Gómez et al., on the other hand, proposed a microfluidic platform with a spatially separated cultivation and measurement chamber. The integration of four sensors allowed multiplexed measurements of glucose, hydrogen peroxide, oxidative reduction potentials as well as conductivity. The sensor was employed to monitor oxidative stress‐induced changes in MRC‐5 cultures.^121^ While, to the authors' knowledge, no optical glucose sensors have been integrated into OoC platforms until now, Fuchs et al. recently reported a microfluidic device with integrated optical oxygen and glucose sensors that could be connected downstream of existing platforms. The sensor displayed a limit of detection (LoD) of 0.6 mM and was validated with cell culture supernatants.^122^ Heo et al. developed another potentially integrable optical sensor based on glucose‐responsive fluorescent hydrogel fibers. By injecting biocompatible PEG‐bonded PAM hydrogel fibers subcutaneously into mice ears, the authors were able to measure blood glucose concentrations for up to 140 days. Despite the sensor's response lag of 10 +/− 5 min, the setup might be an attractive alternative for measuring neuronal glucose uptake in vitro (Figure 6b).^123^ An alternative solution to integrated glucose sensors are commercially available sensing platforms (Table S4) such as the GlucCell®, a glucose monitoring system capable of determining the glucose concentration in sampled cell culture media within 15 s. Fleeman et al. used this portable device to investigate whether the apolipoprotein E4 variant, a genetic risk factor in AD, influences glucose utilization in astrocytes.^124^

##### Lactate

Predominately produced by astrocytes in response to neuronal activity, lactate not only serves as an energy source for neurons but has been shown to modulate neuronal function as well as BBB development and integrity.^125^, ^126^ Furthermore, as lactate is produced via aerobic glycolysis, together with glucose, it constitutes an important analyte for monitoring metabolism‐associated changes in NDDs. As demonstrated in the previous section, electrochemical lactate sensors have already been successfully incorporated into multisensor‐integrated OoC platforms (Table S2). In addition to integrated sensors (Figure 6a), several promising off‐chip sensing strategies have been reported (Table S3). For example, Weltin et al. developed a dipstick electrode for monitoring lactate and oxygen concentrations in cell culture supernatants. While the sensor was not able to detect the oxygen consumption of spheroids due to high reoxygenation rates, changes in lactate secretion were successfully monitored in response to the two compounds antimycin A and bosentan (Figure 6i).^127^ Cordeiro et al. reported an amperometric sensor for the simultaneous in vivo measurement of cerebral glucose, lactate, and pyruvate levels. The authors functionalized Pt wires with a permselective membrane and analyte‐specific oxidases. The sensor was able to monitor changes in brain metabolism upon administration of either glucose or insulin.^128^ A similar in vivo sensing approach was published by Booth et al. The sensor was functionalized with lactate oxidase to measure lactate concentrations in mice brains via the amperometric response to the enzymatic byproduct hydrogen peroxide.^129^ In contrast to electrochemical sensors, however, the number of optical lactate sensors that have been validated for cell culture applications is limited. One example of a potential optical sensor for OoC applications has been presented by Zheng et al., who developed a nanoprobe capable of determining extracellular lactate concentrations on a single‐cell basis. The measurement mechanism relies on the enzymatic conversion of lactate into pyruvate and the simultaneous reduction of NAD^+^ to NADH, which subsequently is measured via fluorescence.^130^ Finally, Matthiesen et al. recently employed a commercially available multiplexed glucose and lactate biosensor to investigate differences in the metabolic turnover of fetal and iPSC‐derived astrocytes.^131^ Commercially available lactate sensors are listed in Table S4. While these monitoring systems usually are employed in point‐of‐care diagnostics, they might be a readily available alternative for the evaluation of lactate levels in OoC setups. For a comparative analysis of the Lactate Scout, Lactate Pro, and Lactate Plus, please refer to Tanner et al.^132^

#### 2.3.2 Strategies for monitoring synaptic and neuronal network dysfunction

Synaptic failure and neuronal network dysfunction precede neuronal loss in numerous NDDs including AD, PD, HD, as well as ALS.^47^, ^48^ Under physiological conditions, synaptic function is tightly regulated and modulated by changes in neurotransmitters, calcium levels, cytoskeletal adaptations, pre‐synaptic vesicle dynamics as well as post‐synaptic signaling. To meet the high energy demand required for maintaining calcium homeostasis and ionic balance neurons rely on optimal mitochondrial performance, linking metabolic and network dysfunction. Excitotoxicity, an important mechanism in the etiology of ALS, mediated by excess glutamate, for example, results in neuronal death via elevated calcium influx and mitochondrial impairment.^47^, ^134^, ^135^, ^136^, ^137^ The role of synaptic dysfunction in NDDs is further supported by the synaptic involvement of key proteins affected by disrupted proteostasis (e.g., α‐syn in PD, huntingtin in HD, tau and Aβ in AD) as well as by proteins encoded by genes known to be involved in NDDs.^138^, ^139^, ^140^, ^141^, ^142^ To that end, next to electrophysiological activity monitoring key neurotransmitters (e.g., dopamine, glutamate) and ionic imbalances enables the assessment of neuronal network dysfunction in microphysiological systems. Furthermore, it serves as an ideal basis to non‐invasively assess successful modulation of neurotransmission and thus symptomatic improvement in vitro.

##### Dopamine

The loss of dopaminergic neurons and the subsequent depletion of dopamine in the nigrostriatal pathway is a key hallmark of PD, making it an important analyte for monitoring pathological alterations associated with the synucleinopathy in vitro.^143^ Kruss et al. developed a high‐resolution imaging sensor for monitoring the dopamine efflux of cells within microfluidic devices. The platform utilizes single‐walled and chemically modified carbon nanotubes, luminous in the near‐infrared, coated onto glass surfaces. Using this sensor, the authors were able to map dopamine release hotspots on cell surfaces and evaluate dopamine release anisotropy due to the spatial resolution of the nanosensor arrays. It has to be noted, however, that dopamine measurements were conducted in PBS, pointing at potential interferences from cell culture media constituents such as ascorbic acid. Furthermore, the authors reported potential interferences from catecholamine homologs such as epinephrine and norepinephrine.^144^ Li et al. proposed an optical sensor utilizing tetrapodal‐shaped‐ZnO microparticles, characterized by a dopamine‐dependent quenched autofluorescence under physiological microenvironments. The authors embedded the tetrapodal‐shaped‐ZnO microparticles with neuroblastoma cells in an alginate/pluronic F127 ink and generated a 3D cell‐laden scaffold using a bioprinting approach (Figure 6c). While the sensor displayed a high selectivity to dopamine compared to ascorbic acid, glucose, and glutamine, interference studies with catecholaminergic neurotransmitters are missing. However, the ease of integration associated with the reported approach makes it highly valuable for the non‐invasive monitoring of catecholamine release in OoC platforms.^145^ Due to the neurotransmitter's electroactive properties, significant research has been conducted in the field of electrochemical dopamine detection alongside the development of optical approaches. One such example is presented by Senel et al., who developed a microfluidic chamber with an integrated three‐electrode setup for the amperometric detection of dopamine. The device was employed to determine dopamine levels in both cerebrospinal fluid and plasma of reserpine‐treated mice, a compound used for inducing PD‐related phenotypes. Comparative analysis revealed lower dopamine levels in reserpine‐treated mice compared to healthy controls, which increased upon treatment with the dopamine precursor L‐DOPA (Figure 6g).^146^ Alternative dopamine sensing approaches include the cultivation of cells on top of cylindrical gold nanoelectrode arrays or interdigitated electrodes, respectively.^147^, ^148^ Abe et al., for example, placed PC12 spheroids on a CMOS‐based chip to create an electrochemical dopamine‐release image.^149^ Senel and colleagues, on the other hand, developed an amperometric dopamine sensor by depositing gold on a PDMS‐based micropyramid array. Neuroblastoma cells were cultivated on top of the electrode array, and dopamine release was monitored upon potassium stimulation. However, no signal increase was observed in the absence of the stimulant.^150^

It is important to note that the sensitivity of electrochemical sensors is dramatically influenced by the measuring matrix and that cell culture media significantly affects sensor performance. For example, a signal loss of ~70%–80% has been reported when adding 10% serum to RPMI medium.^151^, ^152^ While the studies focused on the analysis of interferon‐γ, it emphasizes the importance of sensor characterization in cell culture medium. Similarly, Zanetti et al. reported a sensitivity decrease of 80%–90% upon changing the sample matrix from PBS to cell culture medium. Nevertheless, the authors were able to detect significant differences in the supernatant of control and PD patient‐specific human midbrain organoids using a redox cycling‐based sensing approach coupled with mercaptopropionic acid modification. Comprehensive interference studies and a comparative analysis with LC‐MRM‐MS were performed to underline the sensor's applicability to monitor alterations in dopamine levels within cell culture medium.^153^ Overall, electrode modification strategies aimed at enhancing sensitivity and selectivity for dopamine sensors have been extensively studied.^154^, ^155^ However, it must be noted that these sensors were primarily validated using PBS or artificial cerebrospinal fluid.

Furthermore, several dopamine sensors have been developed for real‐time monitoring in vivo. Due to their application in complex biological matrices, the sensors may be applicable to monitor dopamine in OoC platforms.^156^, ^157^ To illustrate, Spitz et al. recently successfully employed a sensor functionalization protocol published by Njagi et al.^157^ to modify screen‐printed carbon electrodes and utilize them as insert‐based sensors for dopamine measurements in a midbrain organoid‐on‐a‐chip platform.^31^ Commercial dopamine sensors may be a viable alternative to overcome the challenges associated with complex electrode fabrication in dopamine sensing. To the best of our knowledge, however, only one commercially available sensor has been reported in the literature. Yavich et al. employed the nafion‐coated carbon fiber to record the catecholamine release in the locus coeruleus of mice in vivo.^158^

##### Glutamate

Glutamate is the main excitatory neurotransmitter in the human brain. After the release of the neurotransmitter into the synaptic cleft, excitatory amino acid transporters take up excess glutamate to prevent excitotoxic effects. Glutamate is mainly taken up by astrocytic transporters, where it subsequently enters the glutamate‐glutamine cycle.^159^ Due to the neurotoxic effects associated with glutamate, imbalances in its homeostasis have been linked to neuronal damage as well as cell death and are considered to play a role in several NDDs, including AD, PD, HD, and ALS.^160^ To that end, glutamate constitutes an important analyte to consider in the context of neurobiological OoC studies. While no glutamate sensors have been integrated into OoC platforms until now, several promising off‐ and on‐chip sensing strategies have been published. Hughes et al., for example, developed an electrochemical biosensor that can be inserted into the wells of a tissue culture plate (Figure 6h). The sensor utilizes the enzymatic conversion of glutamate to 2‐oxoglutarate to reduce Meldola's Blue, whose subsequent oxidation generates a current response. The sensor was successfully employed to monitor the response of HepG2 cells to paracetamol in real time over a period of 8 h.^161^ In contrast, Nasr et al. directly inserted a microelectrode modified with the enzyme glutamate oxidase into cerebral organoids for glutamate measurements. Using this approach, significant differences in the glutamate levels of dorsal and ventral organoids were observed.^162^ It is important to highlight, however, that this measurement approach is invasive and, therefore, should be considered as end‐point analysis in its current form. Samper et al., on the other hand, developed a 3D‐printed device for the real‐time and online microdialysate analysis of brain injury patients. The device consists of a compartment for the microdialysate and three needle‐based sensors for glucose, lactate, and glutamate monitoring (Figure 6f). The real‐time measurement of the microdialysate of an intensive care unit patient revealed a spontaneous increase in glutamate levels as well as a simultaneous decrease in glucose.^163^ The sensing strategy may thus be utilized for the downstream analysis in OoC models as electrodes can easily be replaced without interrupting long‐term cultivations, recalibration is possible by integrating valves, and device fabrication is simple and inexpensive (Figure 6e). Furthermore, several studies on invasive real‐time glutamate monitoring in vivo using functionalized microelectrodes have been reported.^164^, ^165^, ^166^, ^167^ For instance, Ganesana et al. proposed a Pt wire functionalized with a permselective membrane, glutamate oxidase embedded in a chitosan matrix, and ascorbate oxidase to monitor glutamate without the interference of ascorbic acid. Increased glutamate levels were recorded in vivo upon electrical stimulation. The sensor's stability was tested over a period of 1 week, revealing a signal reduction of 40% upon daily use, while biweekly measurements only decreased the signal by 5% (Figure 6j).^164^ While this sensor provides a promising candidate for OoC applications, the limited lifetimes of enzyme‐based sensors have to be taken into consideration.

##### Ions

As neuronal dysfunction goes hand in hand with altered ion (Na^+^, K^+^, Ca^2+^) fluxes, ion‐selective electrodes might provide another promising tool for monitoring neurodegenerative processes in vitro. As of now, however, ion‐selective sensors are limited to a handful of examples due to challenges associated with the presence of ions in cell culture media and, thus, poor signal‐to‐noise ratios. Taurino et al., for example, combined Pt nanopetals with K^+^‐sensitive membranes to monitor cellular viability as a function of K^+^ release. Continuous monitoring was enabled by integrating the sensor into a separate sensing platform downstream of a microfluidic bioreactor. Changes in cellular viability were observed upon exposing HepG2/C3A cells to DI water and the drug acetaminophen.^168^

#### 2.3.3 Sensing strategies for monitoring disrupted proteostasis

Disrupted proteostasis is considered another key hallmark in NDDs, indicated by the accumulation of ubiquitinated and aggregated proteins (e.g., Aβ, tau, α‐syn, TDP‐43) within the brain.^47^, ^48^ In general, one can distinguish between two major cellular protein degradation mechanisms: (i) the autophagy‐lysosomal pathway and (ii) the ubiquitin‐proteosome system. While the autophagy‐lysosomal pathway mediates the clearance of organelles and protein aggregates, the ubiquitin‐proteosome system is involved in the proteolysis of monomeric and misfolded proteins.^169^ Both mechanisms exhibit impaired functionalities in NDDs. Next to a correlation between genes associated with familial forms of NDDs and the two protein degradation mechanisms (e.g., LRRK2, UBQLN2, Parkin, PSEN), aggregated and post‐translationally modified proteins (e.g., tau, TDP‐43, α‐syn) have been shown to affect protein clearance and enhance seeding propensity in vivo.^170^, ^171^ To that end, Aβ, tau, α‐syn, and TDP‐43 constitute important analytes for monitoring the onset and progression of NDD‐related phenotypes in vitro.

##### α‐synuclein

Under physiological conditions, the protein α‐syn is involved in synaptic vesicle trafficking, neurotransmitter release, and chaperone activity. Aggregated forms of the protein, however, can disrupt normal cellular processes and have been associated with NDDs, including Lewy body dementia, multiple system atrophy as well as PD.^172^, ^173^, ^174^ As such, information on soluble, insoluble, and phosphorylated α‐syn, a post‐translational modification associated with freshly formed aggregates, can provide valuable insights into the onset and progression of disease phenotypes.^175^ Wu et al. developed two electrochemiluminescent aptasensors using different functionalization protocols capable of detecting 19–35 fM oligomeric α‐syn in 1000‐fold diluted serum. Indium tin oxide functionalized glass was either modified with a gold nanoparticle/metal–organic framework composite and a thiolated aptamer or with a metal–organic framework and a carboxylated aptamer, resulting in a detection limit of 0.42 and 0.38 fM, respectively.^176^ While the developed sensors were not validated in cell culture media, the low LoD in the fM range renders the sensing approach promising for OoC applications. Lee et al., on the other hand, employed peptide‐imprinted polymers to measure soluble α‐syn in 1000‐fold diluted cell culture supernatants of human midbrain organoids. Comparative analysis of control and PD‐specific organoids carrying a triplication mutation in the α‐syn gene revealed significantly lower levels of soluble α‐syn in patient‐derived organoids. Results were supported by immunofluorescence analysis.^177^ So‐called molecularly imprinted polymers (MIPs) are widely employed as molecular recognition elements in electrochemical sensors. However, MIPs have also been combined with alternative sensing strategies such as fluorescence‐, surface plasmon resonance (SPR‐), colorimetric‐, and electrochemiluminescence‐based methods to confer analyte specificity. MIP‐based sensors have gained growing interest over the last years due to their low costs, ease of manufacture, high selectivity as well as sensitivity. A recently published manuscript by Adampourezare et al. reviews MIP sensors in the context of NDDs‐relevant markers.^178^

##### Amyloid‐β

Aggregated forms of the peptide Aβ have been associated with several NDDs categorized under the term amyloidoses. In fact, the ratio of the two isoforms Aβ40 to Aβ42 in cerebrospinal fluid has emerged as an important diagnostic marker for AD, as Aβ42 has been shown to be reduced by approximately 50% in AD patients.^4^ Several reviews have focused on the latest developments in the field of Aβ sensors, however, sensor‐integrated AD models remain scarce.^179^, ^180^, ^181^ Rushworth et al. developed an impedimetric Aβ sensor utilizing a cellular prion protein fragment as a biorecognition molecule. The sensor revealed significantly reduced levels of oligomeric Aβ in the supernatant of β‐secretase inhibitor IV‐treated 7PA2 CHO cells.^182^ Similarly, using the cellular prion protein, Zhao et al. detected elevated Aβ oligomer levels in cerebral protein extracts from AD mice compared to control samples.^183^ A comprehensive overview of aptamer‐based sensors is given by Zamanin et al. However, included sensors were primarily validated in serum or (artificial) cerebrospinal fluid.^181^ Zhou et al., for example, developed an electrochemical aptasensor for the detection of oligomeric Aβ in artificial cerebrospinal fluid. No response to monomeric or fibrillar Aβ was observed.^184^ *You* et al. published an aptamer‐based MIP sensor for the detection of Aβ oligomers in human serum. The biosensor exhibited a linear range between 0.005 and 10 ng/mL and an LoD of 1.22 pg/mL.^185^ By employing multi‐walled carbon nanotubes and delaminated titanium carbide MXene in an electrochemical MIP sensing approach, Özcan et al. detected Aβ42 in human plasma samples with an LoD of 0.3 fg/mL.^186^ Gagni et al. reported an optical approach employing an ELISA‐based assay immobilized onto silicone substrates to detect Aβ42 in human cerebrospinal fluid samples. The protocol employed fluorescence read‐out and resulted in an LoD of 73 pg/mL in artificial cerebrospinal fluid.^187^

##### Tau protein

Tau is a microtubule‐associated protein essential in maintaining the cytoskeletal structure and stability of neuronal cells. Abnormal protein accumulation and aggregation have been linked to several NDDs, including AD, where the presence of neurofibrillary tangles is considered one of the key pathological features. While aggregated tau predominately occurs intracellularly, several studies have confirmed its extracellular presence, underlining its applicability as non‐invasive monitoring parameter.^188^ Razzino et al. developed a promising amperometric immunosensor (LoD: 1.7 pg/mL) using modified screen‐printed carbon electrodes capable of measuring tau protein levels in plasma and brain tissue extract samples. Comparative analysis of extracted samples from healthy and AD patients revealed elevated tau levels under pathological conditions.^189^ For further information on tau‐specific sensors, please refer to Phan et al., who summarized recent developments in the field of Aβ and tau biosensors.^180^

##### TDP‐43

TDP‐43 is an RNA/DNA‐binding protein that is involved in the regulation of RNA processing. Disrupted proteostasis characterized by the accumulation of TDP‐43 aggregates is associated with several NDDs including ALS as well as AD.^190^ As of now, however, the development of sensors for TDP‐43 remains scarce. Recently, Wallace et al. demonstrated the label‐free detection of TDP‐43 in a buffer by electrochemical impedance spectroscopy.^191^ Similarly, Dai et al. developed a label‐free biosensor based on differential pulse voltammetry, capable of detecting TDP‐43 in human serum.^192^ In contrast, Serafin et al. labeled captured TDP‐43 with horseradish peroxidase (HRP) and subsequently measured substrate conversion by amperometry, resulting in an LoD of 12.8 pg/mL. The developed sensor revealed elevated TDP‐43 levels in the plasma of AD patients.^193^

#### 2.3.4 Sensing strategies for monitoring neuroinflammation and oxidative stress

Neuroinflammation accompanied by microgliosis as well as astrogliosis constitutes another key hallmark shared among NDDs.^47^, ^48^ Upon activation, microglia, the resident macrophages of the brain, secret inflammatory cytokines (e.g., TNFα, IL‐1β, and IL‐6) and chemokines (CCL2), produce ROS, and initiate phagocytosis.^194^ Microgliosis is initiated by aggregated proteins, synaptic dysfunction as well as ROS, and—if left unresolved—ultimately results in neuronal cell death. In concert with microglia, activated astrocytes release cytokines and chemokines as well, thus further contributing to synaptic dysfunction, imbalances in energy homeostasis, protein aggregation, as well as neurodegeneration.^195^ Neuroinflammation is closely intertwined with oxidative stress, another common hallmark of NDDs. Imbalances in ROS and reactive nitrogen species result in DNA damage, post‐translational modification of proteins, enhanced protein aggregation propensity, and neuronal apoptosis. It has to be noted that neurons are particularly vulnerable to oxidative stress due to high numbers of polyunsaturated fatty acids in cell membranes, high metabolic activity as well as low antioxidant defense mechanisms.^196^

In summary, monitoring both cytokine release as well as oxidative stress via, for example, ROS and reactive nitrogen species within microphysiological systems constitutes an ideal basis for investigating the two critical NDD hallmarks neuroinflammation and oxidative stress in vitro.

##### Cytokines and reactive oxygen species

As neuroinflammation is known to contribute to NDDs, cytokines constitute important analytes to consider when monitoring disease onset and progression in vitro.^197^ Furthermore, cytokines have already been successfully detected in OoC platforms using both optical (e.g., SPR, luminescence) and electrochemical sensing strategies. In general, SPR‐associated techniques are based on the interactions of light with conductive electrons, resulting in the generation of so‐called surface plasmons (Figure 5d). While SPR focuses on the propagation of the generated plasmons, localized SPR monitors changes in localized plasmons.^198^ As cellular adhesion, or the adsorption of molecules (e.g., cytokines) onto the surface, induces alterations in the generated plasmons, SPR‐based strategies show great promise for non‐invasive monitoring in in vitro systems. Zhu et al., for example, developed a biomimetic adipose‐tissue‐on‐chip with a multiplexed localized SPR‐based sensor for the simultaneous detection of four cytokines (Figure 5e). Antibody‐conjugated gold nanorods were employed for the real‐time measurement of the pro‐inflammatory cytokines IL‐6 and TNF‐α and the anti‐inflammatory cytokines IL‐10, and IL‐4.^67^ Abdullah et al. employed a fluorescence sandwich ELISA to optically detect cytokines in neural cultures. While the approach is limited by long analysis times of 4 h, up to 10 cytokines can be detected simultaneously by employing a multi‐in situ tagging strategy.^199^ Matharu et al., on the hand, developed an electrochemical aptamer‐based sensor for the detection of cell‐secreted TGF‐β1. To avoid cell proliferation on the electrode surface, actuatable PDMS microcups were integrated into the microfluidic device.^200^ Ortega et al. connected a muscle‐on‐a‐chip device to a sensing unit containing screen‐printed gold electrodes functionalized with IL‐6 or TNF‐α specific capture antibodies (Figure 5c). Increased IL‐6 and TNF‐α secretion was detected upon electrical stimulation or LPS administration, respectively.^133^ Similarly, Pui et al. modified electrode surfaces with anti‐TNF‐α antibodies and applied electrochemical impedance spectroscopy for the detection of the cytokine in the supernatant of Jurkat cells.^201^ Finally, Song et al. reported a needle‐shaped sensor for the in vivo detection of IL‐8 using electrochemical impedance spectroscopy, applicable to insertion‐based approaches.^202^ For a comprehensive overview of electrochemical biosensors in cytokine detection, please refer to Dutta et al.^203^

Reactive oxygen species (e.g., O_2_˙^–^, H_2_O_2_, HO˙) are linked to the onset of NDDs by contributing to oxidative stress, neurodegeneration as well as inflammation. As such, they constitute another key analyte to be monitored in microphysiological systems.^204^ For instance, Li et al. developed a microfluidic platform comprised of a cell cultivation chamber and four downstream microchannels for the detection of reactive oxygen species (H_2_O_2_) and reactive nitrogen species (ONOO^−^, NO˙, NO_2_^−^), respectively. In detail, increases in reactive oxygen and nitrogen species were monitored using Pt‐black electrodes after stimulating RAW 264.7 macrophages with a calcium ionophore. Measurements were conducted in LockeX1 buffer (pH: 7.4).^205^ In contrast, Matharu et al. developed a ROS sensor by covering a gold electrode with an HRP‐PEG‐hydrogel to catalyze and subsequently measure the reduction of H_2_O_2_ within a microfluidic device. Cyclic voltammetry measurements were employed to monitor H_2_O_2_ levels of primary rat hepatocytes in response to ethanol treatment with and without pre‐incubation of antioxidants.^206^

## 3 CONCLUSION

To summarize, over the last few years, significant progress has been made in the development of OoC models for studying NDDs and identifying potential therapeutic intervention strategies. While published models have not yet reached the degree of physiological mimicry required for preclinical studies, essential groundwork has been laid to foster further advancements in the field. Important biological aspects that are still lacking include the co‐presence of (a) the BBB, (b) the meningeal lymphatics, (c) immune‐relevant cell types, as well as (d) isogenic cell sources. Once such intricate and complex brain models have been developed, it is imperative to address their robustness, reproducibility, and scalability, which serve as an essential prerequisite for achieving predictive validity. Moreover, the additional combination of multiplexed sensing strategies with OoC technology needs standardization and assay harmonization to be accepted by industry and regulatory agencies alike. To date, the majority of published on‐chip sensors monitoring key microenvironmental parameters, crucial metabolites, as well as tissue‐specific functionalities and markers, however, are still restricted to proof‐of‐concept studies and have not been taken up by the broader OoC community. The latter might be linked to the highly interdisciplinary nature of OoC technology and its inherent subdivisions, resulting in varying and discipline‐specific inhibition thresholds.

Overall, three sensor integration approaches have been employed in OoC systems, including (i) on‐chip integration, (ii) downstream integration, and (iii) insertion‐based integration. While electrical and optical sensors can generally be integrated directly into existing OoC platforms, challenges related to sensor stability and sensitivity loss make downstream connection a more suitable option for electrochemical sensing methods. Thereby recalibration and sensor regeneration protocols can be conducted without interfering with running experiments. In addition to monitoring microenvironmental parameters, the progressive nature of NDDs highlights the significance of time‐resolved monitoring of pathological phenotypes. This approach becomes particularly important as it can unveil phenotypes that may otherwise be obscured by invasive endpoint analysis. A variety of on‐ and off‐chip sensing approaches applicable to monitoring dynamic changes in NDD‐related analytes have been developed so far, including sensors for studying tissue functionalities such as barrier integrity and electrophysiological activity, metabolites, neurotransmitters, cytokines, biomarkers, and ions. Overall, sensors for tissue functionality (MEA, TEER), key metabolites, and cytokines can readily be integrated into NDD platforms, with detailed protocols being available in the literature. However, it is important to note that apart from dopamine, sensors for other classes of neurotransmitters and biomarkers have not yet been introduced into OoC platforms. Here, one of the major obstacles is matrix‐induced sensitivity loss, which occurs upon switching to more complex matrices such as cell culture media. Media‐induced high signal‐to‐noise ratios also constitute a major drawback in monitoring ion fluctuations within the extracellular space using ion‐selective sensors. However, considerable advances have been made in the field of ion‐sensitive dyes (e.g., Na^+^, K^+^), fluorescent protein labeling, and multi‐modal imaging providing an additional route to non‐invasive ion monitoring in OoC platforms.^207^, ^208^

To conclude, a plethora of on‐ and off‐chip sensors focusing on microenvironmental parameters and key analytes in NDDs have already been developed and can, in principle, be effortlessly integrated into OoC‐platforms, paving the way for next‐generation preclinical models. We hope that this review will help in this endeavor by addressing this—in the authors' point of view—essential and often neglected aspect in the development of reliable OoC platforms and providing a comprehensive overview of current sensor‐integrated OoC devices as well as of promising on‐ and off‐chip sensors capable of monitoring NDD‐associated phenotypes in vitro.

## AUTHOR CONTRIBUTIONS

**Sarah Spitz:** Conceptualization (lead); writing – original draft (lead); writing – review and editing (lead). **Silvia Schobesberger:** Conceptualization (equal); writing – original draft (equal); writing – review and editing (equal). **Konstanze Brandauer:** Conceptualization (equal); writing – original draft (equal); writing – review and editing (equal). **Peter Ertl:** Conceptualization (equal); funding acquisition (lead); writing – review and editing (equal).

## CONFLICT OF INTEREST STATEMENT

The authors declare no conflicts of interest.

**Abbreviations**
AD: Alzheimer's disease
ALS: amyotrophic lateral sclerosis
Aβ: amyloid‐β
BBB: blood–brain barrier
CMOS: complementary metal‐oxide semiconductor
ECIS: electrical cell‐substrate impedance sensing
HD: Huntington's disease
HRP: horseradish peroxidase
iPSC: induced pluripotent stem cell
LoD: limit of detection.
MEA: multi‐electrode arrays
NDD: neurodegenerative disease
OoC: organ‐on‐a‐chip
PD: Parkinson's disease
ROS: reactive oxygen species
SPR: surface plasmon resonance
TEER: trans‐epithelial/endothelial electrical resistance
α‐syn: α‐synuclein

## Supporting information


**Data S1.** Supporting Information.

## Data Availability

Data sharing is not applicable. No new data generated.
