# Supplementary material for: Sensor‐integrated brain‐on‐a‐chip platforms: Improving the predictive validity in neurodegenerative research
Source: Bioeng Transl Med. 2023 Oct 18;9(3):e10604. doi: 10.1002/btm2.10604 (PMC11135156; doi:10.1002/btm2.10604)
Supplement: Supplementary file 1 — Data S1. Supporting Information. [file BTM2-9-e10604-s001.pdf]

Supplementary Information

Sensor Integrated Brain-on-a-Chip Platforms: Improving the Predictive Validity in Neurodegenerative Research

Abbreviations

NDD, neurodegenerative disease; OoC, organ-on-a-chip; AD, Alzheimer's disease; PD, Parkinson's disease; Aβ, amyloid-β; α-syn, α-synuclein; BBB, blood-brain barrier ; TEER, trans-epithelial/endothelial electrical resistance; ECIS, electrical cell-substrate impedance sensing ; iPSC, induced pluripotent stem cell; MEA, multi-electrode arrays; LSPR, localized surface plasmon resonance; MIPs, molecularly imprinted polymers; ROS, reactive oxygen species; LoD, limit of detection; PoC, proof-of-concept; OCR, oxygen consumption rate; β-NGF, nerve growth factor; GDNF, glial cell-derived neurotrophic factor; PDGF-AA, platelet-derived growth factor-AA; CNTF, ciliary neurotrophic factor; IGF-1, insulin-like growth factor; BNDF, brain-derived neurotrophic factor; FGF-2, fibroblast growth factor; NT-3, neurotrophin-3; BMP-4, bone morphogenetic protein-4; ITO, indium tin oxide; CK-MB, creatine kinase-MB; GST-α, gluthathione S-transferase α; AA, ascorbic acid; UA, uric acid; SE, serotonin; L-DOPA, 3,4-dihydroxy-L-phenylalanine; NEP, norepinephrine; DOPAC, dihydroxyphenylacetic acid; EP, epinephrine; Glc, glucose; Lac, lactate; DA, dopamine; AC, acetaminophen; Gly, glycine; Lys, lysine; Glu, Glutamate; CSF, cerebrospinal fluid, HER-2, human epidermal growth factor receptor-2; EIS, electrochemical impedance spectroscopy.

Table 1: *Overview of relevant OoC-integrated sensing strategies. The level of complexity (LoC) is categorized into easy (I), intermediate (II), and elaborate (III) handling, integration, and analysis – categorization does not consider technical equipment requirements.*

Analyte/ Parameter	Sensing Strategy	Modelled Tissue and Dimensionality	Read-out	Application	Note	LoC	Ref.
Barrier integrity	TEER	Skin<br>• Primary human foreskin-derived dermal fibroblasts (HDF)<br>• Primary human epidermal keratinocytes (HEK)<br>• 3D	Real-time In-situ	Assessment of barrier integrity	• PoC<br>• Impaired visualization but improved sensitivity distribution<br>• Electrode wire	I	90
		Blood-retinal-barrier<br>• Human retinal pigment epithelial cell line (ARPE-19)<br>• Primary human endothelial cells (HREC)<br>• Human neural cell line (SH- SY5Y)<br>• 2.5D	Real-time In-situ	Assessment of barrier integrity	• PoC<br>• Parallel multi-chamber setup employing electrodes embedded on one common substrate	II	209
		Intestine<br>• Human epithelial cell line (Caco-2)<br>• 2D	Real-time In-situ	Validation of a mathematical model to account for geometric differences in Transwell® systems and microfluidic devices	• PoC	II	94
		Lung/Intestine<br>• Human epithelial cell line (Caco-2)<br>• Primary human airway epithelial cells<br>• 3D	Real-time In-situ	Assessment of impedance spectroscopy for monitoring TEER	• PoC<br>• Changes in impedance spectra allow for the monitoring of tissue differentiation (villi formation)	II	88,210
		BBB<br>• Primary human endothelial cells<br>• Primary human astrocytes<br>• Primary human pericytes	Real-time In-situ	Assessment of the barrier permeability performance of multi-functionalized gold nanorods	• PoC<br>• Vertically arranged endothelial barrier	II	95

		• 3D			• Planar gold electrodes		
		BBB<br>• Human iPSC-derived endothelial cells<br>• Human iPSC-derived astrocytes<br>• Human iPSC-derived neurons<br>• 3D	Real-time<br>In-situ	Assessment of barrier integrity	• Emulation of physiologically relevant TEER values for up to 5 days	II	39
		BBB<br>• Human iPSC-derived brain microvascular endothelial cells<br>• Primary human astrocytes<br>• Primary human pericytes<br>• 2.5D	Real-time<br>In-situ	Effect of hypoxia on the differentiation of brain microvascular endothelial cells, BBB integrity and molecular transport	• TEER levels similar to those estimated for the human BBB *in vivo*	II	89
		BBB<br>• Primary murine astrocytes<br>• Primary murine pericytes<br>• Primary murine microvascular endothelial cells<br>• 2.5D	Real-time<br>In-situ	Effect of co-culture and TNF-**α** with and without dexamethasone on barrier integrity (12 d)	• PoC<br>• Combination of hydrostatic pressure and paper flow restrictor<br>• Singular lumen model	I	84
		BBB<br>• Primary murine astrocytes<br>• Primary murine endothelial cells<br>• 2.5D	Continuous<br>In-situ	Effect of ECM, co-culture, and histamine exposure on barrier integrity (4 d)	• PoC<br>• Thin film gold electrode arrays	II	87
		BBB<br>• Human iPSC-derived brain microvascular endothelial cells<br>• 2.5D	Inserted	Comparative analysis of dextran perfusion and TEER data	• PoC<br>• Commercial OrganoPlate®<br>• Ag/AgCl electrodes	I	91
		BBB<br>• Murine cerebral endothelial cells (bEnd.3)<br>• Epithelial-like glioblastoma cell line (U-87)<br>• 3D	Inserted	Barrier integrity	• PoC<br>• 3D-printed model (2-photon polymerization)	I	78
	ECIS	BBB<br>• Human iPSC-derived brain microvascular-like endothelial cells<br>• Human iPSC-derived astrocyte-like cells<br>• 2D	Real-time<br>In-situ	Effect of the peroxynitrite generator linsidomine and the combinatorial addition of the antioxidant N-acetylamide on barrier integrity	• Interdigitated gold electrode on polycarbonate membrane<br>• OSTE+ device	II	50
		Placenta<br>• Human trophoblast cell line (BeWo)<br>• 2D	Continuous<br>In-situ	Comparison of TEER and ECIS/ Assessment of barrier integrity upon exposure to nanoparticles (ZnO_2 and SiO_2)	• PoC<br>• Interdigitated gold electrode on membrane	II	97
	Voltammetry	Endothelium<br>• Primary porcine aortic endothelial cells<br>• 2D	In-situ	Assessment of barrier integrity	• Electroactive tracer<br>• Membrane-based setup<br>• Electrodes integrated into bottom channel	II	96
Cellular viability	Impedance spectroscopy	Cancerous tissue<br>• Human epithelial-like cell lines (HepG2, HeLa)<br>• 3D	Continuous<br>In-situ	Effect of three chemotherapeutic drugs on the cell index in 2D and 3D (embedded in Matrigel) culture settings (96 h)	• Vertical gold electrodes	I	211
		Cerebrum<br>• Human neural stem cell-derived spheroids<br>• 3D	Real-time<br>In-situ	Monitoring neurodegenerative effects upon the addition of Aβ	• Optical accessibility (ITO electrodes)	II	212
Oxygen	Luminescence	Liver<br>• Human epithelial-like cell line (HepG2) [63]<br>• Primary rat hepatocytes [64]<br>• 2D	In-situ	Establishment of a cell-mediated oxygen gradient results in partial liver-like zonation	• Oxygen-sensitive material is embedded in a polystyrene layer<br>• Intensity imaging	I	65,66
		Liver<br>• Bipotent progenitor cell line (HepaRG)<br>• Peripheral blood mononuclear cells<br>• Primary endothelial cells (HUVECs)<br>• 3D	In-situ<br>Continuous	Monitoring the OCR in static and dynamic cultivation setups	• Spray coated oxygen sensor at the inlet and the outlet of each chamber	I	213
		Epithelium/Connective tissue<br>• Human epithelial-like cell line (HeLa)<br>• Normal human dermal fibroblast (NHDF)	In-situ<br>Continuous	Validation of planar oxygen sensor films for cell culture	• PoC	I	214

		• 2D/3D			• Ratiometric intensity imaging<br>• Tested with 2D and 3D cell cultures<br>• Temporal and spatial resolution		
		Liver<br>• Primary rat hepatocytes<br>• Primary rat liver sinusoidal endothelial cells<br>• Primary rat non-parenchymal cells<br>• 3D	In-situ	Development of a perfusable multiwell bioreactor with oxygen probes to measure OCR at different flow rates	• Fluorescence lifetime<br>• Sensing layer of the oxygen probe is submerged into the cell culture medium	I	60
		Epithelium/Connective tissue/ Endothelium<br>• Human lung carcinoma epithelial-like cell line (A549)<br>• Primary human fibroblasts (NHDF)<br>• Human adipocyte-derived stem cells<br>• Primary endothelial cells (HUVECs)<br>• 2D/3D	In-situ Continuous	Investigation of the OCR of 2D and 3D hydrogel-based cultures	• Fluorescence lifetime<br>• Impact of microfluidic materials, coatings, cell densities, cell types and flow rates on oxygen levels	I	64
		BBB<br>• Immortalized murine brain endothelial cell line (cerebEND)<br>• 2D	In-situ Continuous	Modulation of oxygen concentrations in microfluidic devices to investigate ischemia	• Fluorescence lifetime	I	70
	Amperometry	Liver<br>• Primary human hepatocytes<br>• Primary rat hepatocytes<br>• 2D	In-situ	Monitoring of respiratory activity after drug administration	• Inkjet-printed sensors<br>• Electrodes on porous membrane<br>• Gradient monitoring (3 sensors in a row)<br>• 8 hours	II	111
Cytokines	LSPR	Adipose tissue<br>• Murine precursor adipocyte cells (3T3L1)<br>• Murine macrophages (J7)<br>• 2D	In-situ	Label-free, high-throughput, and multiplexed cytokine secretion analysis to monitor adipose tissue inflammation	• Antibody-conjugated Au nanorods specific to IL-6, TNF-a, IL-10, and IL-4<br>• Range of detection: ~10-10 000 pg/mL	II	67
	Luminescence	Neural rosettes<br>• Human iPSCs-derived embryoid bodies<br>• 2D/3D	In-situ	Assessment of ten cytokines using a multiplex in-situ tagging technology based on sandwich ELISA	• Fluorescence intensity<br>• Tested cytokines: **β**-NGF, GDNF, PDGF-AA, IL-6, CNTF, IGF-1, BDNF, FGF-2, NT-3, BMP-4<br>• LoD: 2.9- 57.2 pg/mL	III	199
	Amperometry	Skeletal muscle<br>• Murine myoblast cell line (C2C12)<br>• 3D	Downstream	Cytokine release as a response to electrical or biological stimulation	• Antibody-based immunoassay<br>• LoD: 8 ng/ml (IL-6), 2 ng/ml (TNF-α)	III	133

Table 2: *Overview of relevant multi-sensor integrated OoC approaches. The level of complexity (LoC) is categorized into easy (I), intermediate (II), and elaborate (III) handling, integration, and analysis - categorization does not consider technical equipment requirements.*

Analyte/ Parameter	Sensing Strategy	Modelled Tissue and Dimensionality	Read-out	Application	Note	LoC	Ref.
Albumin, CK-MB, GST-α, pH, oxygen	EIS Absorbance Luminescence	Heart/Liver<br>• Human embryonic stem cell-derived cardiomyocyte organoid<br>• Primary human hepatocytes or human hepatocellular carcinoma cell line (HepG2) spheroids<br>• 3D	Downstream	Study cardio- and hepatotoxic effect of doxorubicin and acetaminophen	• Antibody-based immunoassay<br>• Regeneration protocol<br>• Dual-organ platform<br>• Additional integration of temperature and flow sensors	III	74,215

Barrier integrity, electrophysiological activity	TEER<br>MEA	Heart<br>• Primary human endothelial cells (HUVECs)<br>• Human iPSC-derived cardiomyocytes<br>• 2D	Real-time<br>In-situ	Integration of a dual sensing approach in previously established OoC platform	• PoC<br>• Pt-black modified gold thin film electrodes	II	49
Barrier integrity, oxygen	TEER<br>Luminescence	Intestine/Kidney<br>• Primary human colon epithelial cells<br>• Proximal renal tubule epithelial cells (hRPTEC)<br>• Dermal microvascular endothelial cells<br>• 2D	Real-time<br>In-situ	Drug-screening/ Assessing the effect of fluid shear stress and co-culture on barrier integrity	• Various cell types and assays tested on platform	II	216,217
Barrier integrity, ROS, pH	TEER<br>Amperometry<br>Absorbance	Liver/Kidney/Lung<br>• Human hepatocellular carcinoma cell line (HepG2)<br>• Human foreskin fibroblast cell line (Hs68)<br>• Human lung adenocarcinoma cell line (NCI-H1437)<br>• Human immortalized proximal tubule epithelial cell line (HK-2)<br>• 2D	Continuous<br>In-situ	Identification of drug-mediated cytotoxic effects/ Effect of ECM and TGF-**β**1 on cell growth, albumin production, and urea release	• PoC<br>• Transparent ITO electrodes<br>4 h ROS measurement	II	218-221
Glc, Lac	Amperometry	Intestine<br>• Human colorectal carcinoma cell line (HCT116)<br>• 3D	In-situ<br>Continuous	Montioring of medium-induced changes in metabolism	• Enzymatic approach<br>• Hanging-drop chip<br>• Plug-in sensor	I	117
Glc, hydrogen peroxide, oxidation-reduction potential, conductivity	Voltammetry<br>Amperometry<br>Potentiometry<br>Conductometry	Lung<br>• Human fibroblast-like cell line (MRC-5)<br>• 2D	Downstream	Monitoring H_2O_2-induced oxidative stress	• Enzyme-based (Glc)<br>• Reactivation and recalibration of sensor possible<br>• On-chip	II	121
Oxygen, pH	Luminescence	Epithelium/Endothelium<br>• Human lung carcinoma cells (A549)<br>• Human intestine cell line (Caco-2)<br>• Primary human endothelial cells (HUVECs)<br>• 2D	In-situ	Monitoring the cellular oxygen uptake and the extracellular acidification rate after the addition of nanoparticles	• Sensor spots<br>• Luminescent lifetime	I	58,71
Oxygen<br>pH	Luminescence<br>Absorbance	Skin<br>• Human dermal fibroblasts<br>• 2D	Downstream	Measuring pH and oxygen for up to 3 days	• Oxygen via fluorescence intensity<br>• pH measurement via absorbance (phenol red)<br>• pH range: 6.5-8	I	56
Oxygen,<br>Glc, Lac	Luminescence<br>Amperometry	Liver<br>• Human hepatocellular carcinoma cell line (HepG2/C3A)<br>• 3D	In-situ (Oxygen)<br>Downstream (Glc, Lac)	Monitoring mitochondrial respiration by real-time measurement of oxygen consumption[61] and real-time analysis of early mitochondrial stress in liver organoids [66]	• Enzymatic approach<br>• Integrated and automated calibration<br>• Replacement of sensor without interruption of experiment<br>• Tissue-embedded microprobes	II	61,62
Oxygen, Glc, Lac	Amperometry	Breast cancer<br>• Human breast cancer stem cell line 1<br>• 3D	In-situ (Oxygen)<br>Downstream (Glc, Lac)<br>Continuous	Monitoring changes in metabolic activity after the administration of Antimycin A or doxorubicin	• Enzymatic approach (Glc, Lac)<br>• On-chip<br>• Long-term continuous monitoring (1 week)<br>• LoD: 7.6 µM (Glc), 6.1 µM (Lac)	II	118
Oxygen, Glc, Lac<br>pH	Amperometry<br>Potentiometry	Glioblastoma<br>• Human fibroblast-like cell line (T98G)<br>• 2D	In-situ (Oxygen, pH)<br>Downstream (Glc, Lac)<br>Continuous	Monitoring changes in metabolic activity after Cytochalasin B administration	• Enzyme-based (Glc, Lac)<br>• pHEMA membrane (Glc, Lac)<br>• Stop-flow	II	119

Oxygen,<br>DA,<br>electrophysiological activity	Luminescence<br>Amperometry<br>MEA	Midbrain<br>• Human iPSC-derived midbrain organoids<br>• 3D	In-situ (Oxygen)<br>Downstream (DA)<br>In-situ (MEA)	Identification of PD-associated phenotypes	• Enzymatic approach (DA)<br>• Oxygen sensor stable for 7 weeks<br>• Neurites guided onto 2D planar MEA using interstitial fluid flow	II	31

Table 3: *Overview of* *promising on- and off-chip sensing strategies applicable to NDD research*. *Ease of integration (EoI) is categorized on a scale from + to +++.*

Analyte/ Parameter	Sensing Strategy / Sensor	Tissue Type, Matrix, Dimensionality	Read-out	Application	Note	EoI	Ref.
Barrier integrity	EIS	*In vitro*<br>• Medium<br>• Human lung cancer cell line (Calu-3)<br>• 2D	In-situ	Assessment of barrier integrity after sodium dodecyl sulfate addition	• PoC<br>• Optical accessibility<br>• Carbon electrodes functionalized with IrOx particles	+++	222
Electrophysiological activity	MEA	*In vitro*<br>• Tyrode's solution<br>• HUES9 and HES3 human embryonic stem cells-derived cardiac organoids<br>• 3D	In-situ	Monitoring the electrophysiological activity of cardiac tissue	• Flexible MEA<br>• Wrapped around tissue<br>• Potential application as a sensor for monitoring biomolecules	+	223
	MEA	*In vitro*<br>• Medium<br>• Human cardiomyocyte cell line (HL-1)<br>• Human iPSC-derived cardiomyocytes<br>• 2D	In-situ	Assessment of intra- (after electroporation) and extracellular activity / Selective drug delivery	• PoC<br>• Selective intracellular transport combined with MEA recording	+	224
	MEA	*In vitro*<br>• Medium<br>• Primary rat neuronal and glial cell populations<br>• 2D	In-situ	Assessment of neuronal activity on network, cellular and subcellular levels	• 26 400 platinum microelectrodes	+	102
	3D MEA	*In vitro*<br>• Hydrogel<br>• Human iPSC-derived neurons<br>• Human iPSC-derived neurons<br>• 3D	In-situ	Non-invasive monitoring of electrophysiological activity	• PoC<br>• Polyimide with Pt black	++	103
	3D MEA	*In vitro*<br>• Human iPSC-derived spinal cord organoids<br>• 3D	In-situ	Non-invasive monitoring of electrophysiological activity with and without optical and chemical stimulation	• Needle-based sensors inserted into organoid<br>• Capable of optical stimulation and drug administration	++	104
Oxygen	Amperometry	*In vitro*<br>• Medium<br>• Human breast cancer cell line (T-47D)<br>• 2D	Continuous	Monitoring of cellular respiration	• PoC<br>• Integrated in cell culture flask<br>• 1 week of monitoring without recalibration<br>• LoD: 0.2 µM	++	225
Oxygen,<br>pH	Amperometry<br>Potentiometry	*In vitro*<br>• Medium<br>• Human pluripotent stem cell-derived cardiomyocytes<br>• 2D	Continuous	Studying changes in metabolism based on carbon source in medium	• PoC<br>• Transwell® setup<br>• O_2 and pH monitored with the same electrode (ruthenium oxide)	+	226
Oxygen,<br>pH	Luminescence	*In vitro*<br>• Medium<br>• Mouse fibroblast cell line (L929)<br>• 2D	In-situ<br>Continuous	Validation of biocompatibility	• PoC<br>• Dual lifetime referencing	+++	227
Glc	Luminescence	*In vivo*<br>• Tissue<br>• Mice (BALB/c Slc-nu/nu)	In-situ	Subcutaneous Glc monitoring for up to 140 days	• Fluorescence intensity<br>• Range: 0-500 mg/dL	+++	123

					• Response lag: 10 +/- 5 min		
	Luminescence	*In vitro*<br>• Medium<br>• Human intestine cell line (Caco-2)<br>• 2D	Downstream<br>On-chip	Sensor characterization	• PoC<br>• Fluorescence lifetime<br>• Enzymatic approach<br>• LoD: 0.6 mM<br>• Requires the presence of an oxygen sensor	+++	122
Glc, ascorbate, Lac	Amperometry	*Ex vivo*<br>• Brain microdialysate<br>• Sprague—Dawley rats	Continuous<br>On-chip	Monitoring metabolic changes in an ischemia/reperfusion model	• PoC<br>• Enzymatic approach<br>• Microfluidic chip<br>• LoD: 200 μM (Glc), 200 μM (Lac), 2 μM (AA)	+++	228
Glc, Lac, choline, Glu	Amperometry	*In vitro*<br>• 20x diluted medium<br>• Human intestine cell line (Caco-2)<br>• 2D	Downstream<br>On-chip	Response to Triton X-100, $CuCl_2$, acetaminophen	• PoC<br>• Enzymatic approach<br>• Simultaneous detection of two analytes<br>• Choline and Glu not tested with cell culture medium	++	229
Oxygen, Glc, Lac<br>pH	Amperometry<br>Potentiometry	*In vitro*<br>• Medium<br>• Murine macrophage cell line (RAW 264.7)<br>• 2D	In-situ<br>On-chip	Development of a multianalyte electrode	• PoC<br>• Stop-flow<br>• LoD and linear range dependent on enzyme concentration and diffusion barrier	+++	120
Lac	Luminescence	*In vitro*<br>• Media<br>• Human cancerous cell lines (HeLa, MCF-7)<br>• Human fetal osteoblasts<br>• 2D	In-situ	Comparative analysis of cancerous and healthy cells	• PoC<br>• Fluorescence intensity<br>• Single-cell analysis<br>• Linear response between 0.06-1mM	++	130
	Amperometry	*In vitro*<br>• Medium<br>• Human epithelial-like cell line (HepG2)<br>• 2D	Downstream<br>On-chip	Monitor response to anti-cancer drug 5-fuorouracil and vincristine sulfate salt	• PoC<br>• Enzymatic approach<br>• Tested interferents (Glc, UA)<br>• LoD: 4.5 μM	+++	230
Lac, oxygen	Amperometry	*In vitro*<br>• Medium<br>• Human hepatic progenitor cell line spheroids (HepaRG)<br>• 3D	Continuous	Response to Antimycin A and Bosentan	• PoC<br>• Enzymatic approach<br>• Inserted biosensor<br>• LoD: 5 - 30 μM (Lac)	+++	127
Lac,<br>pH	Amperometry<br>Potentiometry	*In vivo*<br>• CSF<br>• Mice	In-situ	Monitoring local changes in Lac concentration	• PoC<br>• Range: pH 6.52-7.56<br>• LoD: 19 +/- 7 μM (Lac)<br>• Response time: 67 ± 7 s	++	129
Lac, Glc, pyruvate	Amperometry	*In vivo*<br>• Tissue<br>• Wistar rats	Continuous	Monitoring effects of Glc and insulin administration	• PoC<br>• Multiplexed enzymatic approach<br>• Inserted needle-based sensor<br>• Tested interferents: DA, DOPAC, UA, AA<br>• LoD: < 5 μM (for all biosensors)	+++	128
DA	Luminescence	*In vitro*<br>• PBS<br>• Neuroprogenitor rat cell line (PC12)<br>• 2D	In-situ	Acquisition of spatial DA release profiles	• Fluorescence intensity<br>• Tested interferents: EP, NEP, AA<br>• DA imaging	++	144
	Luminescence	*In vitro*<br>• PBS/Media<br>• Human neuroblastoma cells (SH-SY5Y)<br>• 3D	In-situ	DA detection in 3D neuronal tissues	• PoC<br>• Fluorescence intensity<br>• Alginate/Pluronic F127-based bio-ink with tetrapodal-shaped-ZnO<br>• LoD: 0.137 μM<br>• Linear range: 5 - 1000 μM	++	145

	Amperometry	*Ex vivo*<br>• CSF<br>• Swiss Webster mice	Continuous<br>On-chip	Monitoring of reserpine-treated mice	• PoC<br>• No surface modification<br>• Tested interferents: Glc, Lac, UA, AA<br>• Tested in blood plasma<br>• LoD: 0.1 nM	+++	146
	Amperometry	*In vitro*<br>• High-K^+ buffer<br>• Neuroprogenitor rat cell line (PC12)<br>• 2D	In-situ	Monitoring of DA release as response to L-DOPA and reserpine	• PoC<br>• Separately cultivated spheroids measured on electrode<br>• Electrochemical imaging via sensor array (400 electrodes)<br>• LoD: < 5 μM	+	149
	Amperometry	*In vitro*<br>• Buffer<br>• Human neuroblastoma cells (SH-SY5Y)<br>• 2D	Downstream	Monitoring of DA release	• PoC<br>• Au-coated arrays of micropyramids<br>• No surface modification<br>• KCl stimulation<br>• Tested interferents: Glc, AA, UA<br>• LoD: 0.5 nM	++	150
	Redox-cycling	*In vitro*<br>• Medium<br>• iPSC-derived midbrain organoids<br>• 3D	Downstream	Monitoring of DA release with and without treatment with LRRK2 inhibitor 2	• Tested interferents: NEP, L-DOPA, EP, DOPAC, GABA, AA<br>• Validated using LC-MRM-MS	++	153
	Amperometry	*In vivo*<br>• Tissue<br>• Male Sprague-Dawley rats	Continuous	Response to electrical stimulation with and without nomifensine	• PoC<br>• Enzymatic approach<br>• Tested interferents: AA, UA, SE, L-DOPA, NEP, EP, DOPAC	++	157
Glu	Amperometry	*In vitro*<br>• Medium<br>• Human epithelial-like cell line (HepG2)<br>• 2D	Continuous	Response to paracetamol	• PoC<br>• Enzymatic approach<br>• LoD: 1.2 μM (PBS), 4.2 μM (cell culture medium)	++	161
	Amperometry	*In vivo*<br>• Tissue<br>• Sprague-Dawley rats	Continuous	Response to electrical stimulation	• PoC<br>• Enzymatic approach<br>• Inserted microelectrode<br>• Functionalization with glutamate and ascorbate oxidase<br>• Tested interferents: SE, adenosine, DA, Glc, UA, AA<br>• LoD: 0.044 μM	+++	164
	Amperometry	*In vivo*<br>• Tissue<br>• Mice (C57BL/6)	Continuous	Response to visual stimulation	• PoC<br>• Enzymatic approach<br>• Inserted needle-based sensor<br>• Tested interferents: AA, UA, AC<br>• LoD: 16 nM	+++	167
	Amperometry	*In vitro*<br>• Tissue<br>• Human cerebral organoids<br>• 3D	In situ	Monitoring Glu release	• PoC<br>• Enzymatic approach<br>• Inserted microelectrode<br>• Tested interferents: Gly, AA, UA, DOPAC, Lys<br>• LoD: 5.6 μM	+++	162
Glu, Glc, Lac	Amperometry	*Ex vivo*<br>• Brain microdialysate<br>• Human	Continuous	Identification of depolarisation events	• Enzymatic approach<br>• Needle-based sensor<br>• Continuous monitoring for 4 days with automated calibration every 2 hours<br>• LoD (Glu): 0.25 μM	+++	163
Glu, Lac	Amperometry	*In vivo*<br>• Tissue<br>• Sprague Dawley rats	Continuous	Monitoring the effects of Glu injections	• PoC<br>• Enzymatic approach<br>• Inserted	+++	166

					• LoD: 220 nM (Glu), 2 μM (Lac)		
Ascorbate, Glu	Amperometry	*In vivo*<br>• Tissue<br>• Wistar rats	Continuous	Monitoring the response to Glu and K^+ application	• PoC<br>• Enzymatic approach (Glu)<br>• Inserted microelectrode<br>• Simultaneous detection<br>• LoD: 204 nM (Glu), 0.7 μM (Lac)	+++	165
Aβ oligomer	Impedance	*Ex vivo*<br>• Cerebral protein extract<br>• Healthy and AD mice	Downstream	Detection of oligomeric Aβ	• PoC<br>• Cellular prion protein-based<br>• Tested interferents: monomeric and fibrillar Aβ<br>• Tested in 10% FBS<br>• LoD: 10^{-9} nM	+	183
	Impedance	*In vitro*<br>• Medium<br>• Chinese hamster ovary cells (CHO 7PA2)<br>• 2D	Downstream	Monitoring the effect of the beta-secretase inhibitor **β**IV	• PoC<br>• Cellular prion protein-based<br>• 20 min incubation<br>• Tested interferents: monomeric Aβ<br>• LOD: ~0.5 pM	+	182
	Voltammetry	Artificial<br>• Artificial CSF	Downstream	Detection of oligomeric Aβ	• PoC<br>• Aptamer-based sensor<br>• 3 h incubation<br>• Tested interferents: monomeric and fibrillar Aβ<br>• LoD: 0.45 nM	+	184
	Voltammetry	*Ex vivo*<br>• Human serum	Downstream	Detection of oligomeric Aβ	• PoC<br>• Aptamer-based MIP sensor<br>• Linear Range: 0.005-10 ng/mL<br>• LoD 1.22 pg/mL	++	185
	Voltammetry	*Ex vivo*<br>• Human serum	Downstream	Detection of oligomeric Aβ42	• PoC<br>• MIP sensor<br>• Linear Range: 1-100 fg/mL<br>• LoD: 0.3 fg/mL	++	186
Aβ	Luminescence	*Ex vivo*<br>• CSF<br>• Human	Downstream	Detection of Aβ42 in human CSF	• PoC<br>• Fluorescence intensity<br>• LoD: 73 pg/mL in aCFS	++	187
Aβ, tau	Luminescence	*Ex vivo*<br>• CSF<br>• Human	Downstream	Multiplexed detection of AD biomarkers	• PoC<br>• Wavelength shift<br>• LoD (buffer): 7.8 pg/mL (Aβ42), 15.6 pg/mL (tau)<br>• No distinct linear range	+	231
Tau	Amperometry	*Ex vivo*<br>• Brain tissue extract and plasma<br>• Human	Downstream	Comparative analysis of tau levels in healthy individuals and AD patients	• PoC<br>• Antibody functionalized screen-printed carbon electrode<br>• Tested in plasma	++	189
α-syn	Voltammetry	*In vitro*<br>• Medium<br>• Healthy and PD patient-specific midbrain organoids<br>• 3D	Downstream	Assessment of PD-specific phenotypes	• Molecular imprinted polymers<br>• 1000x diluted supernatants<br>• Sensor regeneration possible<br>• LoD: 65 fM	+	177
	Electro-chemiluminescence	*Ex vivo*<br>• Serum<br>• Human	Downstream	Detection of **α**-syn oligomers	• PoC<br>• Aptamer-based sensor<br>• 1000x diluted serum<br>• LoD: ~0.4 fM	+	176
TDP-43	Voltammetry	*Ex vivo*<br>• Serum<br>• Human	Downstream	Detection of TDP-43 in human serum	• PoC<br>• LoD: 0.5 ng/ml	+	192

TDP-43, tau	Amperometry	*Ex vivo*<br>• Brain tissue extract and plasma<br>• Human	Downstream	Detection of tau and TDP-43 in samples of healthy and NDD-diagnosed patients	• PoC<br>• Enzymatic approach<br>• LoD: 2.3 pg/ml (tau), 12.8 pg/ml (TDP-43)	+	193
Troponin T, CK-MB, HER-2	Impedance	*In vitro*<br>• Medium<br>• Human embryonic stem cell derived cardiac organoids or iPSC-derived cardiac organoids<br>• Human epithelial cell line (SK-BR-3) spheroids<br>• 3D	Downstream	Assessment of cardiotoxic effects of doxorubicin	• PoC<br>• Aptamer-based sensor<br>• Dual-organ OoC platform	++	232,233
ROS, RNS	Amperometry	*In vitro*<br>• Buffer<br>• Murine macrophage cell line (RAW 264.7)<br>• 2D	Continuous<br>Downstream<br>On-chip	Monitoring response to a calcium ionophore	• PoC<br>• Pillars to avoid downstream cell loss	++	205
ROS	Voltammetry	*In vitro*<br>• Buffer<br>• Primary rat hepatocytes<br>• 2D	In situ<br>On-chip	Monitoring response to ethanol exposure and antioxidants	• PoC<br>• Enzymatic approach<br>• LoD: 0.2 µM (H_2O_2)	++	206

Table 4: *<u>Commercially available sensors potentially applicable in NDD research</u>. Ease of integration (EoI) is categorized on a scale from + to +++.*

Analyte/ Parameter	Sensing Strategy / Sensor	Matrix	Read-out	Application	Manufacturer	Note	EoI	Ref.
Oxygen	Luminescence	*In vitro*<br>• Hydrogel<br>• Human epithelial-like cell line (HepG2, HepG2 3A4)<br>• Murine primary hepatocytes	In-situ<br>Continuous	Monitoring the cellular behavior towards acetaminophen when systematically varying dissolved oxygen concentrations	CPOx-beads, ibidi GmbH	• Microsensor beads embedded in a collagen matrix with cells<br>• Fluorescence lifetime	+++	63
Oxygen	Luminescence	*In vitro*<br>• Media<br>• Human colorectal adenocarcinoma cells (Caco-2)<br>• 2D	In-situ	Measuring oxygen levels in various cultivation approaches	PSt3, PreSens Precision Sensing GmbH	• PoC<br>• Fluorescence intensity	+++	234
Oxygen	Luminescence	*In vitro*<br>• Media<br>• Chinese hamster ovary cell line (CHO)<br>• Murine embryonic stem cells<br>• 2D	Downstream	Monitoring cellular responses after inhibiting mitochondrial activity	FTC-PSt3, PreSens Precision Sensing GmbH	• Flow cell<br>• Oxygen uptake rates of different mammalian and stem cell lines<br>• Fluorescence intensity	+++	114
Glc	Amperometry	*In vitro*<br>• Media<br>• Primary mouse neurons and astrocytes<br>• 2D	Downstream	Investigation of the impact of an AD genotype on astrocytic Lac production and Glc metabolism	GlucCell™, CESCO Bioengineering Co.	• Range: 20-500 mg/dL<br>• Portable	n.a.	124
Glc and Lac (Glutamine and Glu)	Amperometry	*In vitro*<br>• Media<br>• Human iPSCs<br>• Fetal cortical astrocytes<br>• 2D	Downstream	Comparative analysis of the metabolic turnover of Lac and Glc in astrocytes derived from human iPSCs and fetal astrocytes	B.LV5, Jobst Technologies	• Flow cell<br>• Linear range:<br>Glc 0.05-25 mM<br>Lac 0.02-15 mM<br>Glutamine 0.04-20 mM<br>Glu 0.04-10 mM	+++	131
Glc or Lac	Amperometry	*Ex vivo*<br>• Whole blood	Downstream	Point-of-care diagnostics	GlukometerPro/ LacPro, BST	• Linear range:<br>Glc 0.05-33.3 mM<br>Lac 0.5-24.0 mM<br>• Portable	n.a.	n.a.
Lac	Amperometry	*Ex vivo*<br>• Whole blood	Downstream	Point-of-care diagnostics	Lac Scout 4, EKF Diagnostics	• Linear range:<br>0.5-25 mM	n.a.	n.a.

						• Portable		
Lac	Amperometry	*Ex vivo*<br>• Whole blood	Downstream	Point-of-care diagnostics	Lac Pro 2 LT-1730, ARKRAY Inc.	• Linear range: 0.5-25.0 mM<br>• Portable	n.a.	235
Lac	Amperometry	*Ex vivo*<br>• Whole blood	Downstream	Point-of-care diagnostics	StatStrip Xpress Lac System, Novo Biomedical	• Linear range: 0.3-20 mM<br>• Portable	n.a.	n.a.
Lac	Amperometry	*Ex vivo*<br>• Whole blood	Downstream	Point-of-care diagnostics	Lac Plus, Lac Plus GmbH	• Linear range: 0.2-25 mM<br>• portable	n.a.	n.a.
Lac	Amperometry	*Ex vivo*<br>• Whole blood	Downstream	Point-of-care diagnostics	THE EDGE, EDGE USA	• Linear range: 0.7-22.3 mM<br>• portable	n.a.	n.a.
ATP	Amperometry	*In vitro*<br>• Balanced salt solution<br>• Human kidney cell line (HK2 cells)<br>• 2D	Downstream	Monitoring connexin hemichannel-mediated ATP release	Sarissa Biomedical/Zimmer Peacock	• Linear range: 1-60 µM	++	236
Glc, Lac, ATP, Ca^{+}, NADH, K^{+} and others	Amperometry	n.a.	Downstream	Medical diagnostics	Zimmer Peacock	• A variety of single-analyte sensors available<br>• Multi-analyte (24-, 96- and 384-well) format possible<br>• Disposable<br>• Expensive	+	n.a.
Catecholamine	Amperometry	*In vivo*<br>• Mice (C57BL/6J)	Inserted	Recording the noradrenalin release in mice brains	CFN30, WPI Inc.	• Nafion-coated carbon fiber<br>• Linear range depends on fiber dimensions	++	158
Glu	Amperometry	*In vivo*<br>• Rats<br>• Mice	Inserted	Monitoring extracelluar Glu levels in rodents	Pinnacle Technology Inc	• Enzymatic approach<br>• Modified with ascorbate oxidase<br>• Linear range up to 50 µM	++	237
